# Identification of putative biomarkers for the serodiagnosis of drug-resistant *Mycobacterium tuberculosis*

**DOI:** 10.1186/1477-5956-10-12

**Published:** 2012-02-25

**Authors:** Lu Zhang, Qingzhong Wang, Wenjie Wang, Yanyan Liu, Jie Wang, Jun Yue, Ying Xu, Wenxi Xu, ZhenLing Cui, Xuelian Zhang, Honghai Wang

**Affiliations:** 1 State Key Laboratory of Genetic Engineering, Institute of Genetics, School of Life Science, Fudan University, 220 Handan Road, 200433 Shanghai, Peoples Republic of China; 2 Shanghai Centre for Clinical Laboratory, 200126 Shanghai, Peoples Republic of China; 3 Shanghai Pulmonary Hospital, Tongji University, 200433 Shanghai, Peoples Republic of China

**Keywords:** Immunoproteomics, *Mycobacterium tuberculosis*, Drug-resistance, Serodiagnosis

## Abstract

**Background:**

Early diagnosis and treatment of *Mycobacterium tuberculosis *infection can prevent most deaths resulting from this pathogen; however, multidrug-resistant strains present serious threats to global tuberculosis control and prevention efforts. In this study, we identified antigens that could be used for the serodiagnosis of drug-resistant *M. tuberculosis *strains, using a proteomics-based analysis.

**Results:**

Serum from patients infected with drug-resistant or drug-susceptible *M. tuberculosis *strains and healthy controls was subjected to two-dimensional gel electrophoresis using a western blot approach. This procedure identified nine immunoreactive proteins, which were subjected to MALDI-TOF-MS analysis. Six recombinant proteins, namely rRv2031c, rRv0444c, rRv2145c, rRv3692, rRv0859c, and rRv3040, were expressed and used to determine the immuno-reactivity of 100 serum samples. Antibody reactivity against rRv2031c, rRv3692, and rRv0444c was consistently observed. Among them, the best sensitivity and specificity of rRv3692 were 37% and 95% respectively. Furthermore, when rRv2031c and rRv3692 or rRv2031c, rRv3692, and rRv0444c were combined in 2:1 or equal amounts, the assay sensitivity and specificity were improved to 56.7% and 100% respectively.

**Conclusions:**

These results suggest that Rv2031c, Rv3692, and Rv0444c are possible candidate biomarkers for effective use in the serodiagnosis of drug-resistant tuberculosis infections, and a combined formula of these antigens should be considered when designing a subunit assay kit.

## Background

More than one-third of the world's population is infected with tubercle bacilli. Poor adherence to tuberculosis (TB) control programs, the subsequent emergence of drug-resistant strains and the widespread prevalence of HIV have contributed to the resurgence of this disease [1]. Multidrug-resistant tuberculosis (MDR-TB) and extensively drug-resistant tuberculosis present serious threats to global TB control efforts. The World Health Organization estimated that the number of new MDR-TB cases in 2004 was 425, 000, and 60% of these cases were located in China, India, and the Russian Federation [2,3].

Most TB-associated deaths are preventable with early diagnosis and treatment [4], and it has been suggested that a quick and accurate tool for early TB diagnosis could save up to 625, 000 lives each year [5]. Currently, optimal diagnostics for TB are a demanding task and new procedures are urgently needed. In endemic areas, sputum-smear microscopy is often the only available and affordable diagnostic test but is only 50-70% sensitive. The diagnostic gold standard is considered to be culturing *Mycobacterium tuberculosis*, as it is sensitive and specific in cases of smear-positive TB. However, results usually take two to four weeks and this test is not routinely used in countries with high prevalence of TB. Recently developed PCR-based tests or interferon-release assays, such as QuantiFERON^® ^TB-Gold, are expensive and require a certain level of expertise [4].

Antibody detection is one of the most common approaches used in the diagnosis of infectious diseases. Antibody production is typically the result of antigen exposure from an infection, and dominant antigens are the primary target for diagnostic and immunoprophylactic therapies. Detection of *M. tuberculosis *antigens released during the active disease stage has been considered a specific and ideal approach in the serodiagnosis of TB because these antigens represent an early disease state [6]. More specifically, antigens including antigen 5 (38 kDa antigen, Rv0934), 27 kDa antigen (MPT51, Rv3803c), 30 kDa antigen (Ag85B, MPT59, Rv1886c), P32 (Rv3804c), 88 kDa antigen (MTB81, Rv1837c), antigen 60 (A60), cord factor, Kp90 and LAM could potentially be used in the serodiagnosis of TB [7]. To date, there are no commercially available serodiagnostic tests for TB. In particular, there is no routine laboratory test for drug-resistant TB with sufficient sensitivity and specificity.

Proteomics is a powerful tool for studying the protein composition of complex biological samples. Protein expression profiles of *M. tuberculosis *under various growth conditions, genetic backgrounds and geographic distribution have been investigated [8-15]. The application of immunoproteomics, combining 2-DE with immunoblotting to find protein candidates for serodiagnostics, is a powerful tool [16-18]. Using this approach, Bassey et al. found some heterogeneity in the responses to *M. tuberculosis *antigens with different patient sera [19]. Karen et al. identified 12 immunogenic proteins from actively replicating H37Rv culture filtrates [20]. However, until recently, there was little information regarding the immunogenicity profiles associated with drug-resistant *M. tuberculosis *strains.

Therefore, the main objective of this study was to evaluate differences between antigen expression and the respective immunoreactivity profiles of drug-susceptible and drug-resistant *M. tuberculosis *strains using an immunoproteomic approach. Identified proteins were subsequently cloned, expressed and purified, and their subcellular location was determined. Antibody reactivity against these proteins was detected by ELISA to evaluate the potential of these antigens to be used as biomarkers for early identification of MDR-TB.

## Results and discussion

### *M. tuberculosis *2-DE profile of whole cell proteins

**Figure 1:**
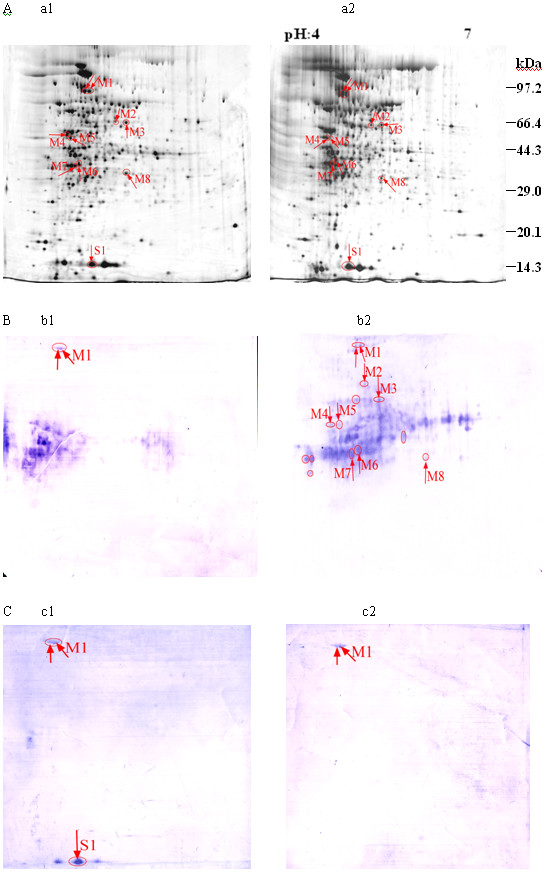
**Comparison of 2-DE analysis and western blot analysis of different *M. tuberculosis *bacterial proteins at pH 4-7**. 80 μg of total protein lysates from *M. tuberculosis *were loaded in the first dimension and approximately 1000 spots were detected after silver staining. Proteins identified were indicated by spot number and in Table 1. (A) 2-DE analysis of cell lysates from antibiotic-susceptible strains (a1), multi-drug resistant strains (a2). (B) Western blot analysis of 2-DE maps of *M. tuberculosis *using pooled serum from patients infected with MDR clinical strains. Protein lysates examined were from sensitive (b1), multi-drug resistant strains (b2). (C) Western blot analysis of 2-DE maps of *M. tuberculosis *using pooled serum from patients infected with antibiotic-sensitive clinical strains. Protein lysates were from sensitive (c1), multi-drug resistant strains (c2). Gels were electroblotted onto PVDF membranes using a semidry transfer unit. All seras were used at the same dilution to 1:400. Labeled protein spots were excised from corresponding 2DE reference gels run in parallel and subsequently identified using MALDI-TOF-MS and MALDI-TOF-MS/MS analysis.

Two-dimensional gel electrophoresis analysis was performed on five MDR clinical isolates and H37Rv, which is a strain that is sensitive to all four first-line TB antibiotics. Representative 2-DE gels of these strains are presented in Figure 1. In the pH range of 4-7, approximately 1000 silver-stained protein spots were reproducibly detected in all strains using the PDQuest version 6.0 software. Three or more gels for each strain were analyzed and compared during the course of this analysis. Only gels with similar normalized volumes for each sample analyzed were selected for western blot analysis.

### Identification of immunoreactive proteins

Proteins from MDR and antibiotic-susceptible strains were examined by western blot for immunoreactivity to serum from patients infected with drug-resistant *M. tuberculosis*. Pooled serums from patients infected with antibiotic-susceptible *M. tuberculosis *were used as negative controls. Figure 1B, C reflect representative antigenic profiles using serum collected from individuals infected with drug-resistant *M. tuberculosis *and drug-susceptible *M. tuberculosis*, respectively. Immunoreactive protein spots selected for further analysis were present on blots probed with patient serum but not visible on blots probed with negative controls. Protein mass fingerprinting of trypsin digest fragments was performed on these proteins, and the results are summarized in Table 1 (including data sourced from Additional files 1, 2, 3, 4, 5, 6, 7 &8). Protein characterization was conducted using MALDI-TOF-MS and MALDI-TOF-MS/MS analysis, and the data were compared with the MASCOT database for sequence matches. Identification of proteins was based on the probability scores for the matches, molecular mass, pI, number of peptide matches and the percentage of the total translated ORF sequence covered by the peptides.

**Table 1:** Immunoreactive protein spots identified by MALDI-TOF-MS

**Spot No^a^**.	NCBI accession no.^b^	**Sanger I.D**.	Protein identification	MW/PI		Mascot score^c^	GRAVY score^d^	2D immunoreactivity		
							
				Theoretical	Experimental			Sensitive *M. tuberculosis*	RIF^r ^*M. tuberculosis*	MDR *M. tuberculosis*
M1	gi|1322434	*Rv1310*	ATP synthase beta chain (atpD)	53093.32/4.76	53061.2/4.86	433, 57	-0.168	S-sera^e^	All sera	All sera

M2	gi|2916918	*Rv0859c*	Acyl-CoA thiolase (fadA)	42413.60/5.14	42387.8/5.2	181, 32	0.035	R-sera	--	--

M3	gi|15610828	*Rv3692*	Methanol dehydrogenase regulatory protein(moxR2)	37903.47/5.51	37904.3/5.43	192, 20	-0.002	--	R-sera	--

M4	gi|15610165	*Rv3028c*	electron transfer flavoprotein (alpha subunit) (fixB, etfA)	31690.02/4.61	31699.8/4.47	746, 68	0.358	--	--	R-sera

M5	gi|2791638	*Rv3040*	Conserved hypothetical protein	31484.26/4.50	31465.7/4.65	98, 6	-0.297	--	--	R-sera

M6	gi|2104333	*Rv2145c*	antigen 84(wag31)	28276.23/4.75	28260.1/4.80	133, 49	-0.726	--	--	R-sera

M7	gi|15607779	*Rv0639*	Transcription antitermination protein(nusG)	25413.54/4.59	25431.0/4.70	175, 23	-0.180	--	--	R-sera

M8	gi|15607585	*Rv0444c*	Conserved hypothetical protein	23882.15/5.82	23883.0/5.81	75, 13	0.062	--	R-sera	--

S1	gi|2896768	*Rv2031c*	Heat Shock Protein hspX (14-kDa antigen)	16226.30/4.90	16217.2/5.0	209, 35	-0.52	--	S-sera	--

a) Spot numbers refer to the numbers on the 2-DE gels showed in Figure 1A-C.

b) Accession no. obtained from the MASCOT results of MALDI-TOF/TOF from the NCBInr database http://www.ncbi.nlm.nih.gov.

c) MASCOT score and percent sequence coverage of all tryptic peptides identified by MALDI-TOF-MS/MS. MASCOT protein scores (based on combined MS and MS/MS spectra (Additional files 1, 2, 3, 4, 5, 6, 7 and 8) greater than 72 were considered statistically significant (p ≤ 0.05).

d) Grand average of hydropathy.

e) Anti-sera from TB patients infected with antibiotic-sensitive *M. tuberculosis*.

f) Anti-sera from TB patients infected with drug-resistant *M. tuberculosis*

**Figure 2:**
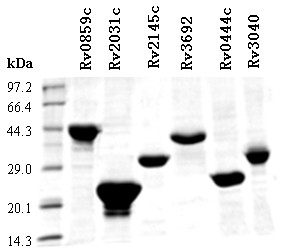
**SDS-PAGE analysis of recombinant proteins**. Rv0859c, Rv2031c, Rv2145c, Rv3692, Rv0444c and Rv3040 were subjected to SDS-PAGE and stained with Coomassie blue. Molecular size markers are indicated on the left in kDa.

**Figure 3:**
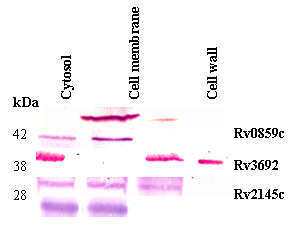
**Subcellular localization of *M. tuberculosis *H37Rv proteins**. Lysates from *M. tuberculosis *H37Rv were fractionated into cytosol (CY), cell membrane (CM), cell wall (CW) and culture filtrate (CF) fractions. These fractions were normalized for total protein content (50 μg) before SDS-PAGE and then electroblotted onto a PVDF membrane and probed with rabbit-antisera raised against Rv0859c, Rv2031c, Rv2145c, Rv3692, or Rv0444c, respectively.

Pooled sera from patients diagnosed with drug-resistant *M. tuberculosis *infection were used to identify immunodominant antigens. The identification of *M. tuberculosis *proteins by human sera suggested that these antigens were expressed in vivo and were capable of eliciting humoral responses. These antigens could therefore be considered attractive immunodiagnostic candidates. A total of nine immunoreactive protein spots (Figures 1, 2) were identified and are reported in Table 1. Among them, the *M. tuberculosis *proteins Rv0639, Rv3028c, Rv3040, Rv2145c, Rv0859c, Rv3692 and Rv0444c were only present when MDR strains were probed with serum from patients infected with MDR *M. tuberculosis*. These proteins were not observed on membranes probed with negative control serum. Rv1310 was identified on all membranes examined. Rv2031c was only present on membranes blotted with drug-susceptible strains and serum from patients infected by antibiotic-sensitive clinical strains (Figure 3).

The nine unique MDR proteins were divided into four functional groups. The first group encompassed metabolic pathway-associated proteins, including carbohydrate metabolism, lipid metabolism, respiratory chain, oxidation-phosphorylation and biotransformation. The functions of these proteins have been well characterized in *M. tuberculosis*, and included ATP synthase beta chain (EC 3.6.3.14) (AtpD), electron transfer flavoprotein (alpha subunit) (FixB, EtfA), acyl-CoA thiolase (EC 2.3.1) (FadA), and hydrodipicolinate reductase (EC 1.3.1.26) (DapB). These proteins represented the major protein spots on the gel. The second group included regulatory proteins whose functions have been biochemically defined, for example: transcription antitermination protein (NusG), methanol dehydrogenase regulatory protein (MoxR2) and Rv0444c, which is the SigK (RskA) regulator of certain *M. tuberculosis *complex members [21,22]. The third group comprised the 16 kDa virulence factor antigen HspX, which is specific to the *M. tuberculosis *complex and is essential to bacilli survival, particularly during latency. This antigen has been used to detect antibody isotypes in sera of TB patients singly or in combination with other antigens [23]. Using the recombinant 38 kDa and 16 kDa antigens for immunodiagnosis provided sensitivities of 59% (culture positive) and 54% (culture negative) and specificity of 98% in patients with pulmonary TB. A recent study suggested that antibody responses to the 16-kDa antigen may be important for the detection of latent TB infections [24,25]. Wag31, whose synonym is Ag84, made up the fourth group of proteins: those associated with cell wall and cell processes. The remaining protein, Rv3040c, is of unknown function. Undefined spots were considered to be either protein mixtures necessitating further separation or proteins present at concentrations below the detection limits of the assay (less than 1 ng).

### Recombinant protein expression and subcellular characterization

Ten recombinant His-tagged H37Rv proteins were cloned and expressed in this study. Six proteins were expressed at high levels both in soluble (rRv2031c, rRv0444c, rRv2145c, rRv3692) and insoluble (inclusion body) aggregates (rRv0859c, rRv3040). As shown in Figure 2, the purified His-tag fusion proteins moved as single bands on the sodium dodecyl sulfate polyacrylamide gel electrophoresis (SDS-PAGE) gels.

Initially, protein hydrophobicity was predicted by calculating the grand average of hydropathy scores using the ProtParam program http://www.expasy.org/tools/protparam.html. A score of > 0.4 indicated a hydrophobic protein likely to be membrane-associated [26]. Using these criteria, all of the identified spots from 2-DE were characterized as hydrophobic (Table 1). The subcellular location of five recombinant proteins was also investigated by western blot analysis using rabbit-antisera raised to the respective proteins. Four different subcellular fractions (culture filtrate, cytosol, cell membrane and cell wall) were analyzed for immunoreactivity. The fidelity of the fractions was confirmed as described previously [27]. Except for Rv2145c, which was present in culture filtrate and cell wall fractions, all proteins were located in the cell membrane of *M. tuberculosis *H37Rv. Several proteins were found in other locations as well as the cell membrane: Rv0859c was present in both cell wall and cell membrane fractions, Rv3692 was identified in the cytosol and cell membrane fractions, Rv0444c was present in the cytosol, cell wall and cell membrane fractions, and Rv2031c was present in the cytosol and cell membrane fractions. These data indicate that most immunogenic proteins detected using the immunoproteomic assay were located in the outer membrane fractions, suggesting that these proteins could function as candidate detection antigens.

### Evaluation of the serodiagnostic potential of the recombinant proteins

**Figure 4:**
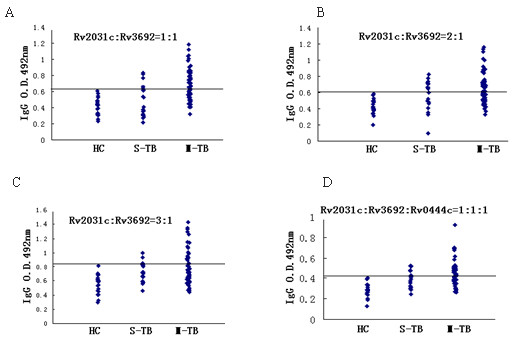
**TB serodiagnose for clinical samples applied with different proteins**. HC, healthy *Mycobacterium bovis *BCG-vaccinated controls; S-TB, pulmonary tuberculosis patients infected by antibiotic-sensitive *M. tuberculosis *strains; M-TB, pulmonary tuberculosis patients infected by multi-drug resistant *M. tuberculosis *strains.Serum reactivity to (A) Rv2031c and Rv3692 at a 1:1 ratio, (B) Rv2031c and Rv3692 at a 2:1 ratio (C) Rv2031c and Rv3692 at a 3:1 ratio and (D) Rv2031c, Rv3692, and Rv0444c at a 1:1:1 ratio.

All recombinant proteins were tested for their potential value in the serodiagnosis of TB by ELISA. Serum from 60 patients infected with MDR *M. tuberculosis *and 20 patients infected with antibiotic-susceptible *M. tuberculosis *was used in the analyses. Only three proteins, rRv2031c, rRv3692, and rRv0444c, were consistently immunoreactive to patient serum and were subjected to further evaluation under various combination strategies, such as rRv2031c and rRv3692 with a ratio of 1:1, 1:2, 1:3, 2:1, 3:1, 3:2, etc. Figure 4 shows the IgG seroreactivity to partial combinations of recombinant proteins at equal or mixed ratios. Combinations of rRv2031c and rRv3692 or rRv2031c, rRv3692, and rRv0444c provided significant levels of seroreactivity when probed with serum taken from drug-resistant *M. tuberculosis *infections, compared with the serum collected from healthy controls. An absorbance of 2 SD (standard deviation) over the mean OD of the healthy controls was considered as the cut-off criterion for seropositivity. Under these conditions, a combination of rRv2031c and rRv3692 at a 2:1 ratio, and the combination of rRv2031c, rRv3692, and rRv0444c with a ratio of 1:1:1, gave a sensitivity of 56.7% and a specificity of 100%, respectively (Table 2 and Figure 4). This indicated that antigens in combination could be potentially used in the serodiagnosis of drug-resistant TB. The use of a single protein in the detection assay showed lower sensitivity and specificity values than combinations of recombinant proteins, further demonstrating the value of multi-component detection formulations. Because the antibody responses to *M. tuberculosis *infections are heterogeneous, the desired sensitivity and specificity in serodiagnostic tests may be achieved by a combination of several proteins, or the fusion of several epitopes into a poly-protein.

**Table 2:** IgG seroreactivity to recombinant *M. tuberculosis *proteins

Recombination proteins		MDR^a^	Susceptible^b^	Healthy controls
		
		Total/Pos(%)	Total/Pos(%)	Total/Pos(%)
Rv0859c		60/14(23.3)	20/1(5)	20/0(0)

Rv2031c		60/20(33.3)	20/2(10)	20/0(0)

Rv2145c		60/13(21.7)	20/0(0)	20/0(0)

Rv3692		60/22(37)	20/0(0)	20/1(5)

Rv0444c		60/15(25)	20/3(15)	20/0(0)

Rv3040		60/7(11.7)	20/2(10)	20/3(15)

Rv2031c:	1:1	60/33(55)	20/5(25)	20/0(0)
	
Rv3692	2:1	60/34(56.7)	20/10(50)	20/0(0)
	
	3:1	60/21(35)	20/7(35)	20/1(5)

Rv2031c:Rv3692:		60/34(56.7)	20/9(45)	20/0(0)

Rv0444c(1:1:1)				

a) Serum from multi drug-resistant *M. tuberculosis *infected patients.

b) Serum from antibiotic-susceptible *M. tuberculosis *infected patients.

Biomarkers of infection disease can indicate normal or pathogenic processes, or pharmacological responses to therapeutic intervention. So biomarkers provide prognostic information for individual patients or cohorts in clinical trials, either about future health status. The need for biomarkers in tuberculosis is most crucial in three areas: to indicate reactivation risk and predict treatment success for patients with latent *M. tuberculosis *infection; to predict durable (non-relapsing) treatment success for patients with active disease; and to indicate protection from tuberculosis by new vaccines for people other than those with active disease [18,28]. Peripheral blood is the most widely used source in clinical practice, although biomarkers can be studied in any tissue or body fluid(including urine, saliva, sputum and breath), Genes, transcripts, proteins, lipids and metabolites can all be measured in blood for biomarker studies [29].

Although this study identified several interesting immunoreactive *M. tuberculosis *proteins, not all could be used for the serological diagnosis of drug-resistant TB. To date we have analyzed immunogenic whole cell proteins that were resolved within the pI range of 4-7. The antigens identified so far largely depended on the combination of the source of the antigens, the immunoreactive immunoglobulin class (IgG or IgM) identified, stage of the diseases and ethnicity of the infected population. The conditions of the immunoproteomic approach (sample denaturation, loss of some proteins under 2-DE conditions) may have impaired the identification of additional antigens. An immunocapture procedure in which antigens could be enriched from subcellular fractions based on their reactivity with immobilized serum from patients infected by MDR could lead to the identification of novel candidate antigens. This kind of approach has already been successfully applied for other diseases [30,31]. It would be of interest to complete the database of potential antigen candidates using non-denaturing and complementary procedures.

## Conclusions

Three candidate antigens, Rv2031c, Rv3692, and Rv0444c, identified in this study appeared to have greater antigenic activity. The antigens were therefore tested in combination using ELISA assays to evaluate the use of these proteins for the serodiagnosis of MDR *M. tuberculosis *infections. The results demonstrated that these antigens, when used in combination, were more effective than when used singly, suggesting that combinations of antigens may yield the desired level of sensitivity without affecting specificity. The validation phase of a diagnostic kit using a combination of rRv2031c and rRv3692 against a large panel of sera from patients infected with drug-resistant *M. tuberculosis *and healthy controls is also in progress. The aim of this work is to supply a commercial serodiagnostic test for drug-resistant TB with sufficient sensitivity and specificity.

To our knowledge, this is the first report that has identified antigen candidates for identifying drug-resistant TB using immunoproteomic analysis. Three candidate protein antigens in combination functioned effectively in the identification of immunoreactive antibodies from patients infected with MDR strains. While the cross-reactivity of the his tag and cleaved purified his-tag free antibody, and cross activity with MTM, are worth to further work to demonstrate.

## Methods

### Bacterial strains and culture conditions

*M. tuberculosis *strain TMC 102 [H37Rv] (ATCC number 27294) was provided by the Chinese Institute for the Control of Pharmaceutical and Biologic Products. Five clinical MDR *M. tuberculosis *isolates from different patients were obtained from the Shanghai Pulmonary Hospital. Their antibiotic susceptibility patterns were determined using the Bactec 960 instrument (Becton Dickinson Microbiology Systems, Sparks, MD, USA). The minimal inhibitory concentrations were determined using a 7H9 solid medium proportional method [32]. Strains were initially identified biochemically and then confirmed by 16S rDNA gene sequencing.

### Human sera

All patient serum samples were obtained from the Shanghai Pulmonary Hospital, China.

Blood samples were drawn from TB patients shortly after diagnosis (culture and/or acid-fast bacillus smear positive and chest X-ray positive). All donors were between the ages of 18 and 65 and provided informed consent.

Serum was collected from patients infected with drug-resistant M. tuberculosis (n = 20) or from patients infected with drug-susceptible M. tuberculosis (n = 20), then randomly divided into four different pools. Each pool included five serum samples and was used for immunoproteomic analysis. Serum from patients infected with drug-resistant M. tuberculosis (n = 60) and drug-susceptible M. tuberculosis (n = 20) was collected for ELISA analysis of IgG reactivity to recombinant antigens. Serum from healthy volunteers was used as a negative control (n = 20). Serum reactivity to recombinant M. tuberculosis antigens were assessed by ELISA as described previously [20].

### Isolation of *M. tuberculosis *proteins

Whole cell extracts were harvested from *M. tuberculosis *during early mid-log growth phase, as described previously [12]. Approximately 10^9 ^bacteria were sonicated in the presence of proteinase inhibitors and then treated with 8 M urea, 2 M thiourea, 70 mM dithiothreitol, 0.5% Biolyte (pH 4-7), 400 μg/mL *n*-octyl-D-glucopyranoside, and 4% CHAPS to completely denature and reduce proteins. Supernatants were then collected after centrifugation at 10 000 × *g *for 15 min. Three subcellular fractions (cell wall, membrane, and cytosolic fractions) of *M. tuberculosis *cell lysates were obtained by differential centrifugation as described previously [12]. Preparation of culture filtrate protein was also performed as described previously [33].

### Two-dimensional gel electrophoresis

Proteins were identified by excision and in-gel trypsinization of respective spots from silver-stained gels as described previously [10]. The analyses were performed using a 4700 MALDI-TOF/TOF Proteomics Analyzer (Applied Biosystems, Foster City, CA, USA) equipped with a 355 nm Nd:YAG laser. Combined MS and MS/MS spectra were submitted to MASCOT version 2.1 (Matrix Science, London, UK) using GPS Explorer software version 3.6 (Applied Biosystems). MASCOT protein scores (based on combined MS and MS/MS spectra) greater than 72 were considered statistically significant (p ≤ 0.05). The individual MS/MS spectrum with statistically significant (confidence interval > 95%) best ion score (based on MS/MS spectra) were accepted. The number of identified peptides, sequence coverage and number of MS/MS identified peptides were considered during protein identification. To eliminate the redundancy of proteins that appeared in the database under different names and accession numbers, the single-protein member belonging to the species Mycobacterium with the highest protein score (top rank) was singled out from the multi-protein family [34]. Isoelectric focusing (IEF) was performed using an IPGphor IEF system (Bio-Rad, Hercules, CA, USA), and 2-DE was performed according to the manufacturer's instructions. Briefly, a 17 cm IPG strip pH 4-7 was rehydrated overnight in rehydration solution (7 M urea, 2 M thiourea, 1% ASB-14, 0.5% Triton X-100, 0.5% 3-10 carrier ampholytes, 55 mM dithiothreitol, 0.002% bromophenol blue) containing 80 μg of proteins in a total volume of 360 μL. Focusing was conducted by stepwise voltage increases as follows: 500 V for 2 hours, 1000 V for 2.5 hours, 8000 V for 5 hours and 8000 V until the total volt-hours reached 80 kVh. Following IEF separation, strips were equilibrated twice (14 min each time) in 8 mL of SDS equilibration buffer (150 mM Tris-Cl, pH 8.8; 6 M urea, 20% glycerol, 2% SDS, 0.002% bromophenol blue) containing 100 mg dithiothreitol and 250 mg iodoacetamide. IPG strips were then placed over a 12.5% vertical polyacrylamide gel (0.75 mm thick, 18 cm wide, and 24 cm long), and electrophoresis was carried out at 30 mA until the bromophenol blue had run to the bottom of the gel. Second-dimension PAGE gels were run in duplicate, with the first gel used for immunoblotting and the second silver stained to serve as a 2-D reference map [35]. Gels were scanned using Molecular Image Fx (Bio-Rad) and analyzed with PDQuest version 6.0 software (Bio-Rad). For each sample, IPG gels were electrophoresed in triplicate on separate occasions. The gel images were normalized according to the total quantity in the analysis set as Griffin TJ [36]. Relative comparison of intensity abundance of differential protein spots was performed with Student's t-test. Any difference with a P-value ≤ 0.05 was considered statistically significant.

### 2-DE western blotting

Proteins subjected to 2-DE gel electrophoresis were transferred onto 0.45 mm Immobilon-P PVDF membranes (Millipore, Billerica, MA, USA) using a semi-dry electro-blotter (Bio-Rad) with Towbin transfer buffer, at a constant current of 1 mA/cm^2 ^for 80 min. PVDF membranes were incubated overnight in PBST (9 mM sodium phosphate, 0.15 M NaCl, and 0.05% v/v Tween 20) containing 5% (w/v) skim milk powder at 4°C with constant rotation. Following three 10-min washes with PBST, blots were incubated for 1 hour at 37°C with human sera diluted 1:400 in PBST containing 2% (w/v) skim milk powder. Human sera from uninfected volunteers and patients infected with drug-susceptible *M. tuberculosis *were used as negative controls. After washing with PBST, blots were incubated with biotinylated anti-human IgG (Vector Laboratories, Burlingame, CA, USA) at a dilution of 1:20, 000 for 1 hour at room temperature. Before and after the addition of the secondary antibody, membranes were washed four times for 15 min in PBST. Membranes were then washed with 50 mM Tris-HCl buffer (pH 7.4) and developed with streptavidin-conjugated alkaline phosphatase (Vector Laboratories). Membranes were then incubated with nitro blue tetrazolium/5-Bromo-4-chloro-3-indolyl phosphate detection substrate. Immunoblotting experiments were conducted in triplicate and no variation was observed between results.

### Tryptic digests and MALDI-TOF-MS

Proteins were identified by excision and in-gel trypsinization of respective spots from silver-stained gels as described previously [10]. The analyses were performed using a 4700 MALDI-TOF/TOF Proteomics Analyzer (Applied Biosystems, Foster City, CA, USA) equipped with a 355 nm Nd:YAG laser. Combined MS and MS/MS spectra were submitted to MASCOT version 2.1 (Matrix Science, London, UK) using GPS Explorer software version 3.6 (Applied Biosystems). MASCOT protein scores (based on combined MS and MS/MS spectra) greater than 72 were considered statistically significant (p ≤ 0.05). The individual MS/MS spectrum with statistically significant (confidence interval > 95%) best ion score (based on MS/MS spectra) were accepted. The number of identified peptides, sequence coverage and number of MS/MS identified peptides were considered during protein identification. To eliminate the redundancy of proteins that appeared in the database under different names and accession numbers, the single-protein member belonging to the species Mycobacterium with the highest protein score (top rank) was singled out from the multi-protein family [34].

### Expression and purification of recombinant proteins and antisera production

The coding sequence for each protein was amplified from *M. tuberculosis *strain H37Rv genomic DNA by PCR. The specific primers included sequences for appropriate restriction sites to enable cloning. The primers and parameters for thermal cycler amplification are shown in Table 3. PCR products were cloned into pET28a or pET30a vectors (EMD Biosciences, San Diego, CA, USA), resulting in the addition of a 6 × His N-terminal leader sequence to each coding sequence. The fidelity of the recombinant plasmid was confirmed by sequencing. Plasmids encoding respective fusion proteins were transformed into *Escherichia coli *BL21 (DE3). Cell cultures were grown to OD_600 _0.6-0.8 in Luria Bertani broth containing 100 μg/mL kanamycin. Protein induction was then carried out by adding 1 mM isopropyl-D-thiogalactoside for 5 hours at 37°C. Induced cells were harvested and washed with cold 50 mM sodium phosphate buffer (pH 7.9) and centrifuged at 8 000 × g for 15 min. The cell pellet was resuspended in Ni-NTA lysis buffer (300 mM NaCl, 50 mM sodium phosphate buffer, pH 7.9) at 50 g/mL wet weight. Bacteria were lysed by sonication and the lysate was centrifuged at 12 000 × g for 30 min. Supernatants were then loaded onto a Ni-NTA His-Binding column (Novagen, Madison, WI, USA) and His-tagged proteins were eluted using elution buffer containing various imidazole concentrations. Pellets were dissolved in lysis buffer in the presence of 8 M urea and then purified using a Ni-NTA His-Binding column as described above. The purified proteins were dialyzed against 5 mM Tris-HCl buffer (pH 7.5) containing 100 mM NaCl and 3% (v/v) glycerol and quantified using Bradford reagent.

**Table 3:** Primers sets for genes amplified by PCR

Gene	Primer sequences(5'-3')	Restriction site used
*Rv1310*	5' CGAATTCATGACTACCACTGCCGAAAAG 3'	EcorI
	5' TATAAGCTTTCACAGCTTGGCGCCGAGAC 3'	HinD III
*Rv3028c*	5' TAGGATCCATGGCTGAAGTACTGGTGCTC 3'	BamHI
	5' TATAAGCTTCTAGCCCTTGCGGGCCTTG 3'	HinD III
*Rv3040*	5' TAGGATCCATGAATTCACCTCGCGAGCC 3'	BamHI
	5' TATAAGCTTTCACAGCGGCCACCCGGTC 3'	HinD III
*Rv2145c*	5' TAGGATCCATGCCGCTTACACCTGCCG 3'	BamHI
	5' TAGAGCTCCTAGTTTTTGCCCCGGTTGAAT 3'	SacI
*Rv0639*	5' TAGGATCCGTGACTACCTTCGACGGTG 3'	BamHI
	5' GACAAGCTTCTAGATCTTGGAGACTTGGCC 3'	HinD III
*Rv3692*	5' TAGGATCCGTGACACAGTCCGCGTCCAAC 3'	BamHI
	5' TGTAAGCTTCTAGCGGGGCACCGGAACC 3'	HinD III
*Rv0444c*	5' TGGATCCATGACTGAACATACCGATTTTGAG 3'	BamHI
	5' TATAAGCTTTCACCCGAGCGGCAGCTCGGC 3'	HinD III
*Rv2031c*	5' TAGGATCCATGGCCACCACCCTTCCCGT 3'	BamHI
	5' GCGAAGCTTTCAGTTGGTGGACCGGATCTG 3'	HinD III
*Rv0859c*	5' ATGGATCCATGTCCGAAGAAGCCTTCATCTAC 3'	BamHI
	5' TATAAGCTTTTAAACCCTCTCGATGATCGTCGC 3'	HinD III

Procedures for antiserum production were based on standard protocols [37]. Briefly, 3-month-old New Zealand White rabbits were immunized with recombinant proteins emulsified with incomplete Freund's adjuvant and boosted four times at 2-week intervals, and antisera collection was performed 7 days after the last boost.

### Enzyme-linked immunosorbent assay

Enzyme-linked immunosorbent assays (ELISA) were used to assess the humoral response in humans to the recombinant proteins identified in this study. Ninety-six-well microtitre plates (Nalge Nunc International, Rochester, NY, USA) were coated with 500 ng of recombinant protein antigen dissolved in 100 μL of 0.05 M carbonic acid buffer, and incubated overnight at 4°C in a humidified chamber. Wells were washed three times with PBST, blocked with 100 μL of blocking buffer (2% bovine serum albumin (w/v) in PBS) for 2 hours at 37°C, and then washed three times with PBST. Sera corresponding to the different clinical groups were diluted 1:400 in blocking buffer and 100 μL was added to each antigen-coated well. Following incubation for 1 hour at 37°C, the plates were washed five times with PBST and then incubated with horseradish peroxidase-conjugated goat anti-human IgG at 37°C for 1 hour. Antibody binding was detected using *o*-phenylenediamine tetrahydrochloride (Sigma Aldrich, Saint Louis, MO, USA) dissolved (1 μL/mL) in citrate-phosphate buffer (pH 5.4) and H_2_O_2_. The reaction was stopped by the addition of 50 μL of 0.5 M H_2_SO_4_, and absorbance values were measured at 492 nm in an ELISA reader (Bio-Rad). Experiments were repeated at least twice with similar results.

The ELISA results were analyzed using cut-off values equal to the mean optical density for the healthy control serum samples plus two standard deviations. Any sample exhibiting an absorbance value above the cut-off value was considered to be positive. For statistical analysis, the differences between groups of TB patients and healthy controls were calculated by the independent-samples *t*-test using the Statistics Package for Social Science (SPSS 13.0, Chicago, IL, USA), with p < 0.05 considered to be statistically significant.

## Competing interests

The authors declare that they have no competing interests.

## Authors' contributions

ZL conceived of the idea for the immunoproteomic study, participated in its design, performed a major portion of the data analysis, and drafted the manuscript; WQZ carried out immunoproteomic analysis and contributed in the preparation of the manuscript; WWJ carried out protein preparation, 2DE, and western blot analysis; LYY assisted in protein sample extraction and western blot analysis; WJ contributed to the bioinformatics analysis; XY carried out ELISA; XWX and CZL participated in the design, implementation, and coordination of the study; ZXL and WHH reviewed the design plan and participated in revision of the final version of the manuscript. All authors read and approved the final manuscript.

## Supplementary Material

Additional file 1 **PMF and MS MS Spectra of Rv0444c**. Click here for file

Additional file 2 **PMF and MS MS Spectra of Rv0639**. Click here for file

Additional file 3 **PMF and MS MS Spectra of Rv1310**. Click here for file

Additional file 4 **PMF and MS MS Spectra of Rv2031c**. Click here for file

Additional file 5 **PMF and MS MS Spectra of Rv2145c**. Click here for file

Additional file 6 **PMF and MS MS Spectra of Rv3028c**. Click here for file

Additional file 7 **PMF and MS MS Spectra of Rv3040**. Click here for file

Additional file 8 **PMF and MS MS Spectra of Rv3692**. Click here for file
