# Supplementary figures and images for: Identification of putative biomarkers for the serodiagnosis of drug-resistant Mycobacterium tuberculosis
Source: Proteome Sci. 2012 Feb 25;10:12. doi: 10.1186/1477-5956-10-12 (PMC3305424; doi:10.1186/1477-5956-10-12)

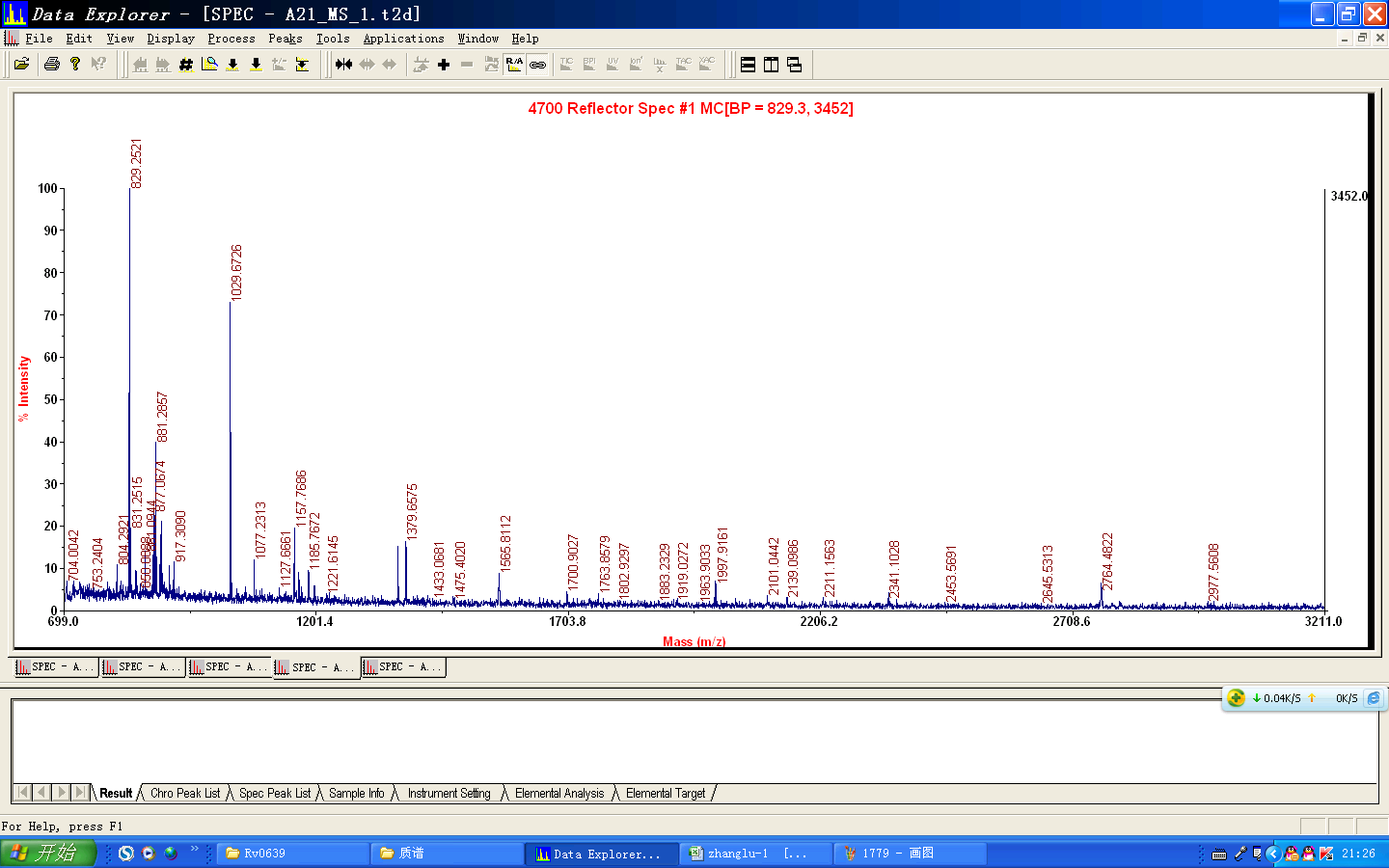


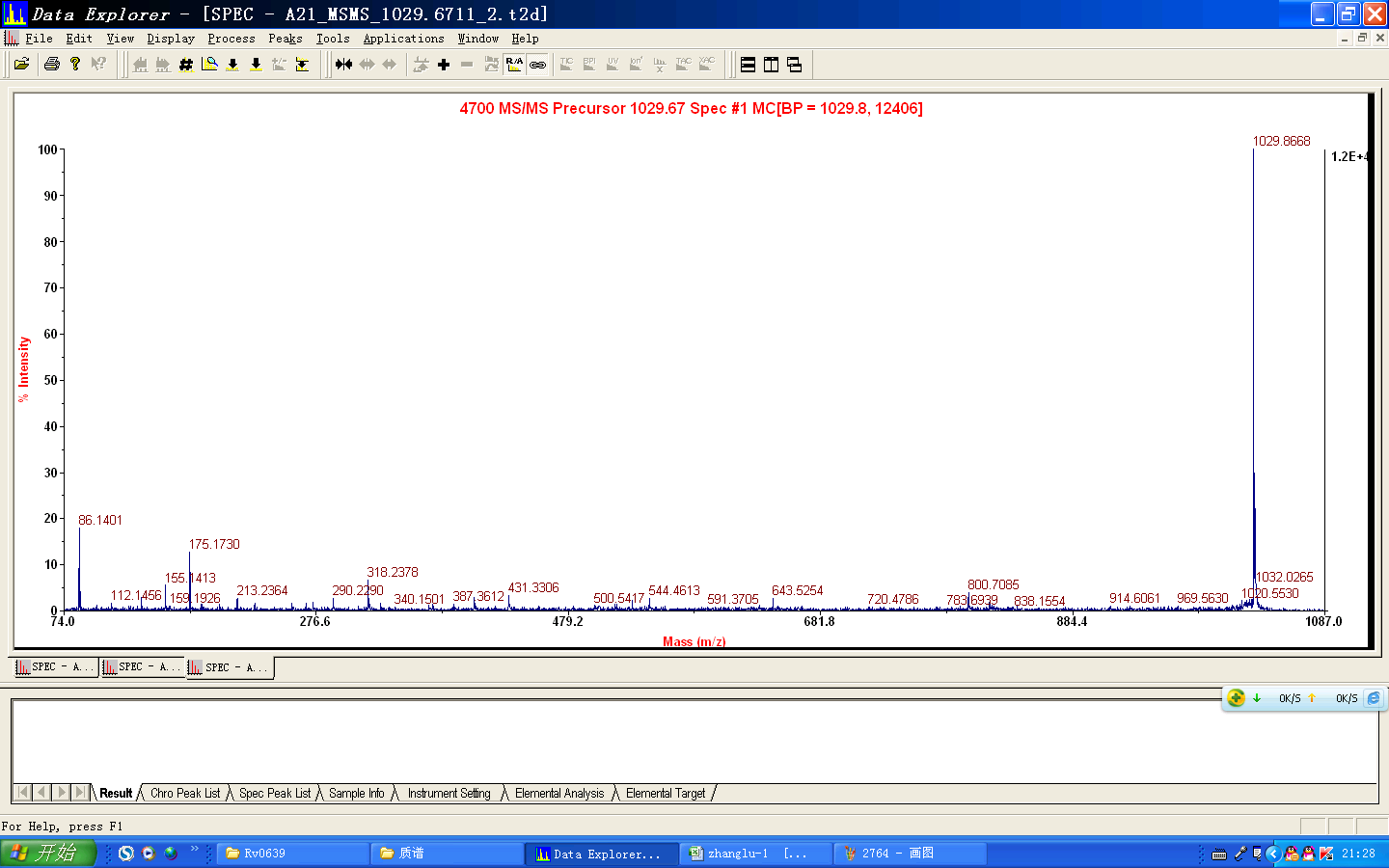


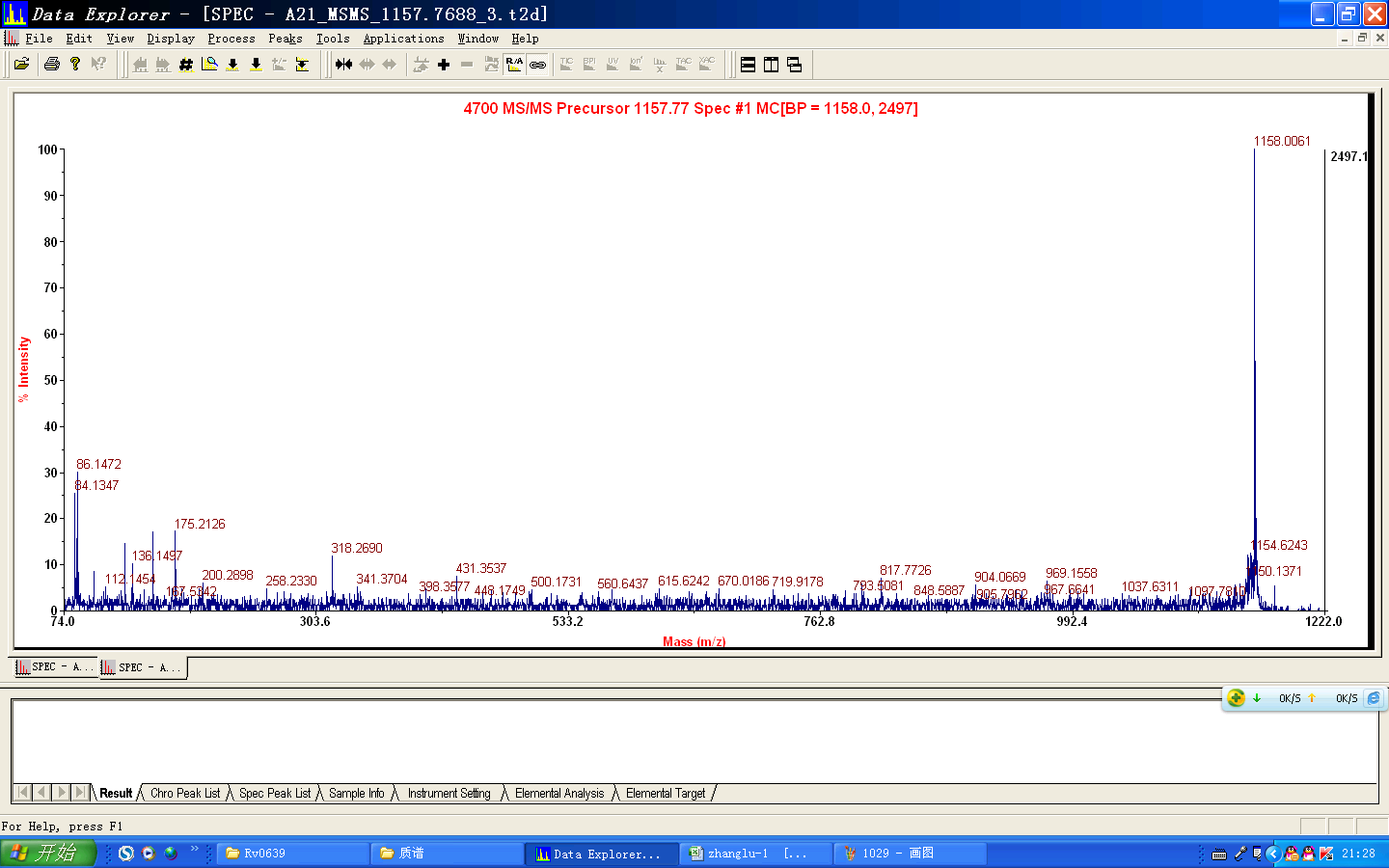


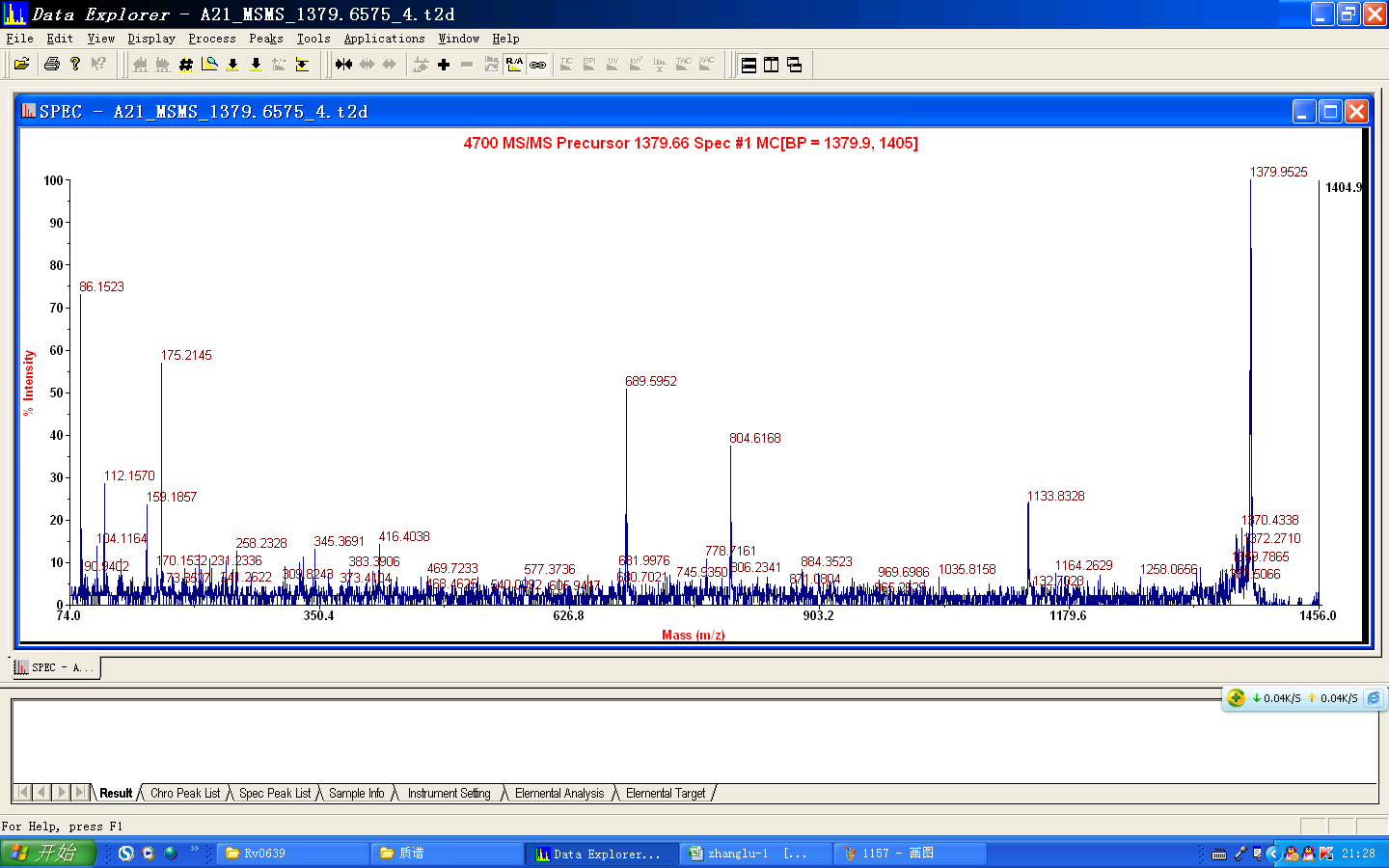


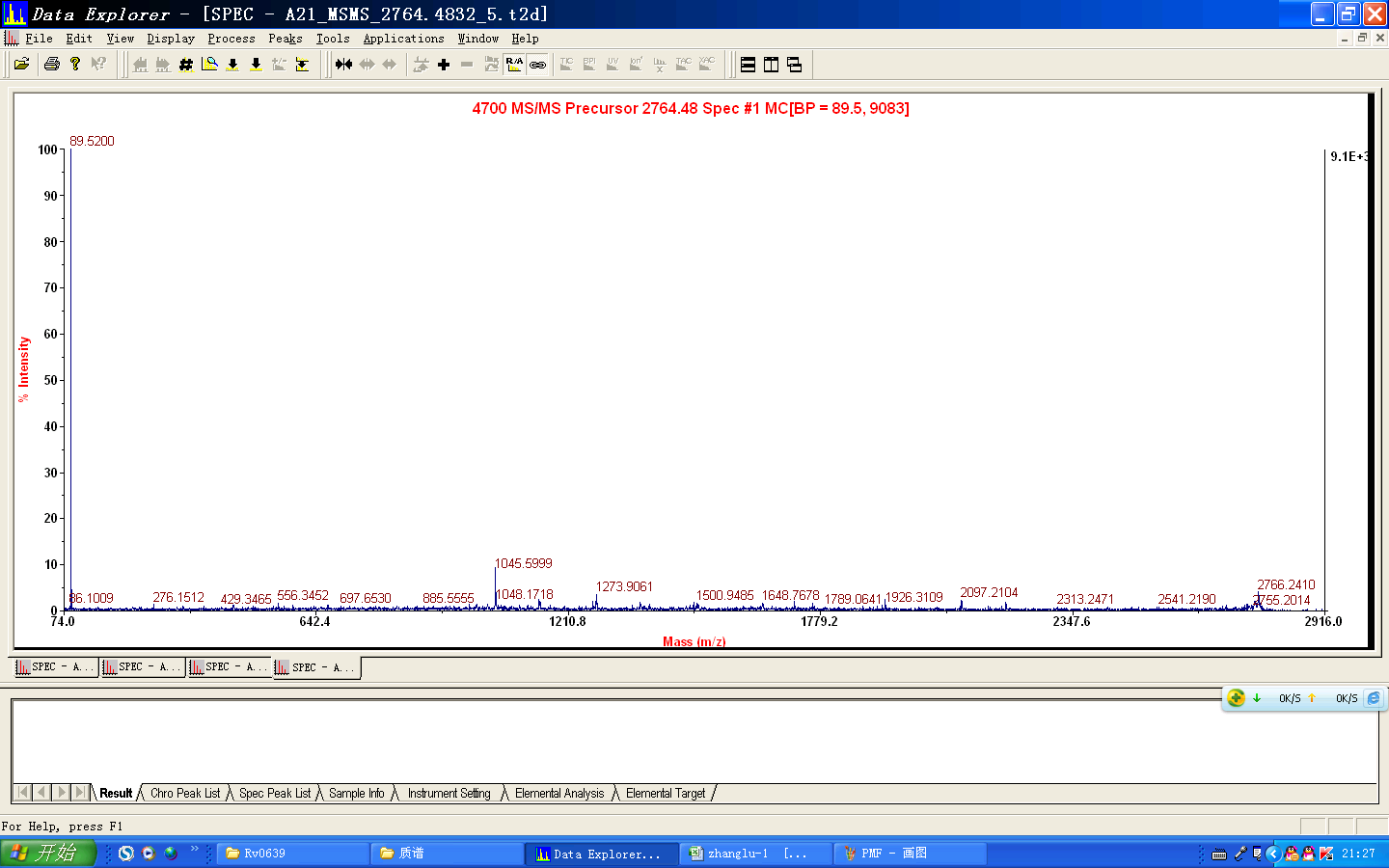

Supplement: Additional file 2 — PMF and MS MS Spectra of Rv0639. [file 1477-5956-10-12-S2.DOC]

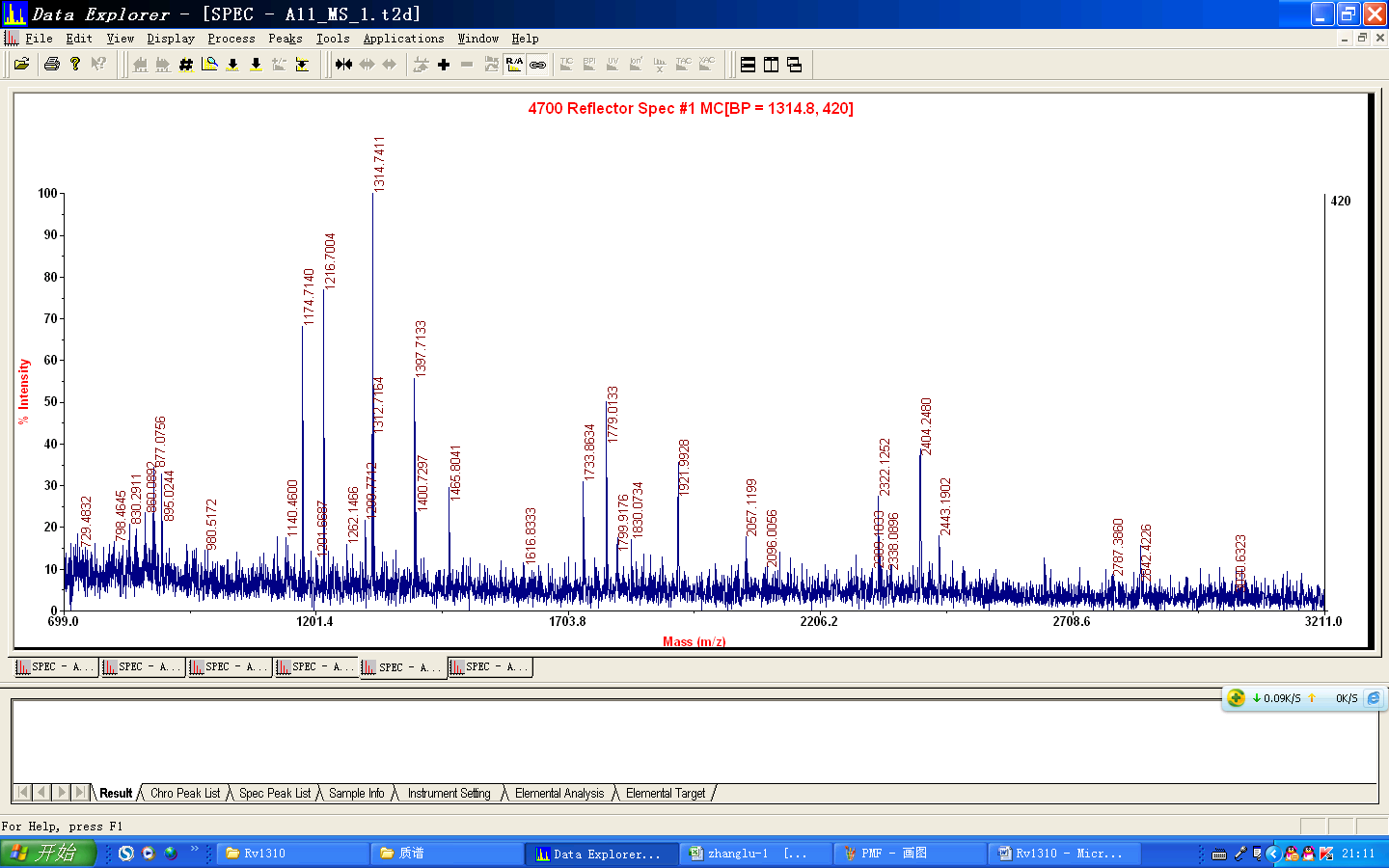


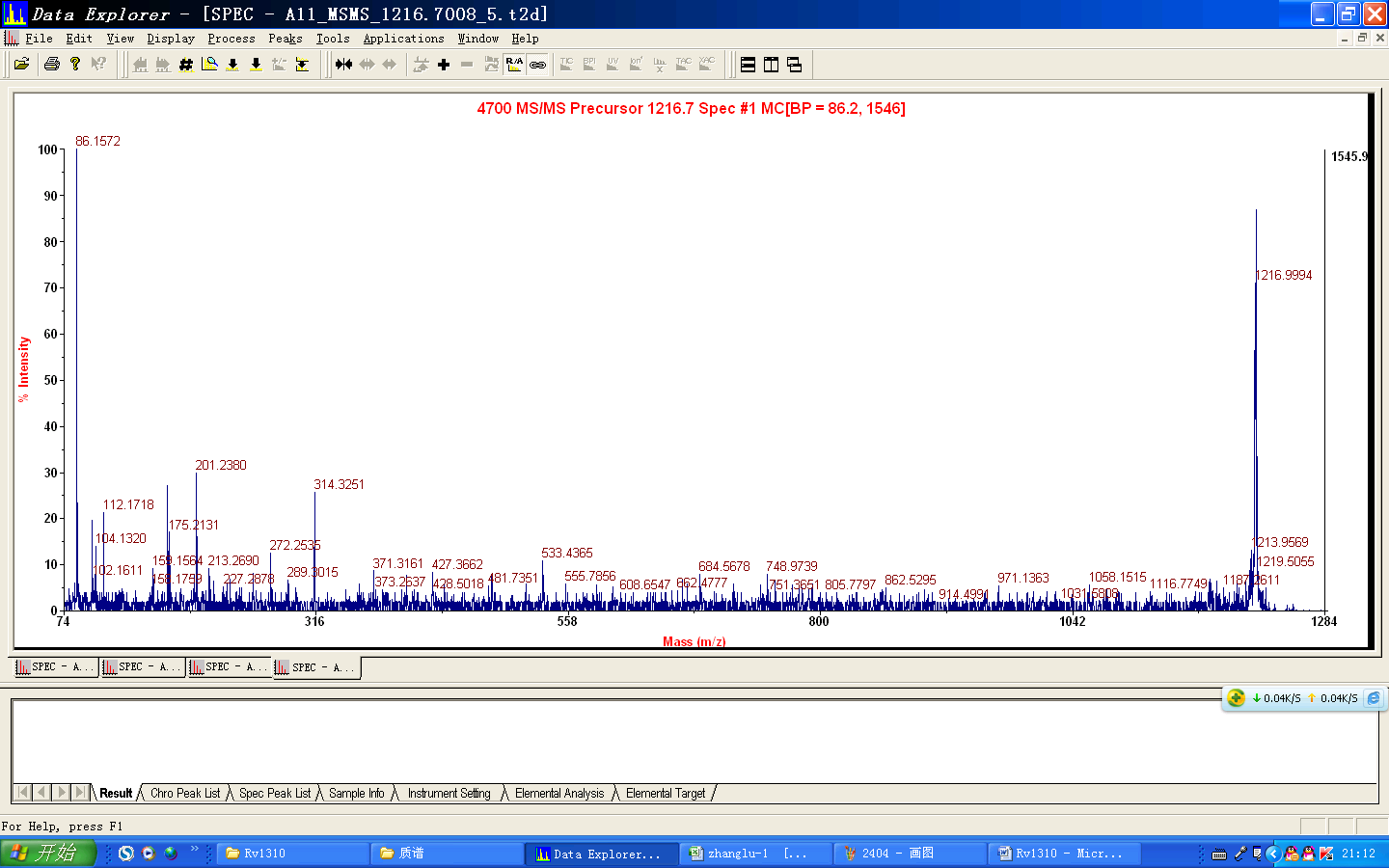


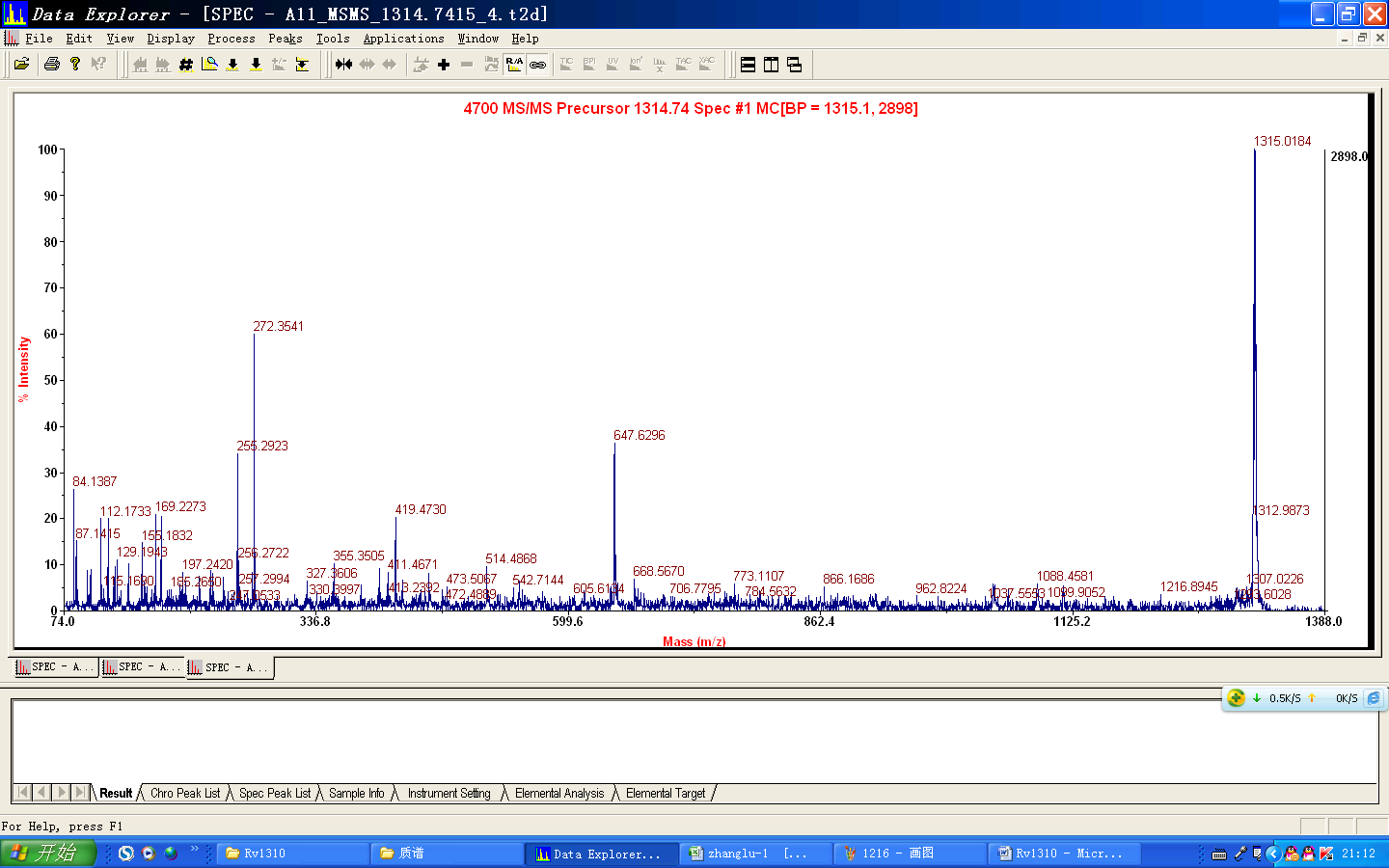


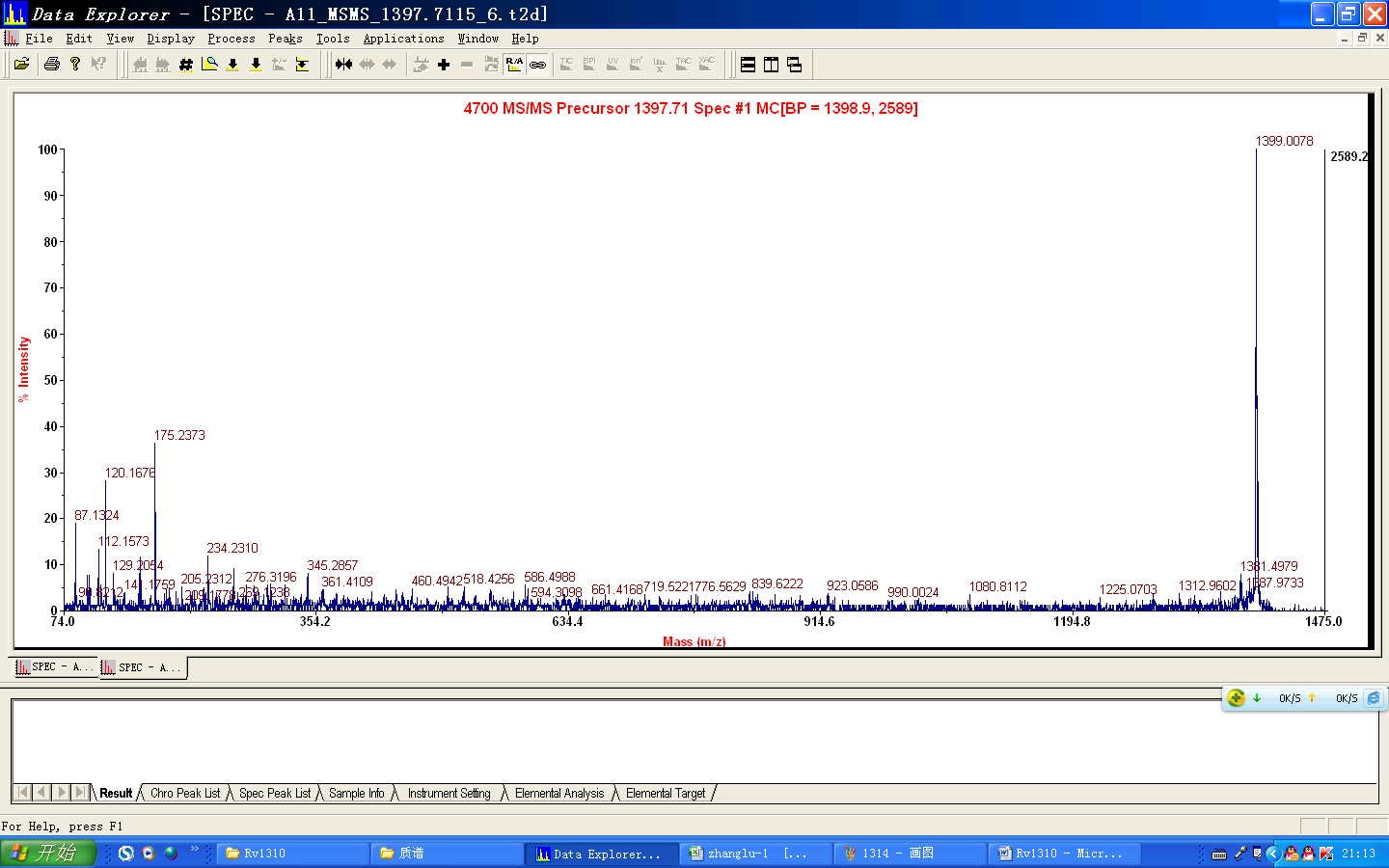


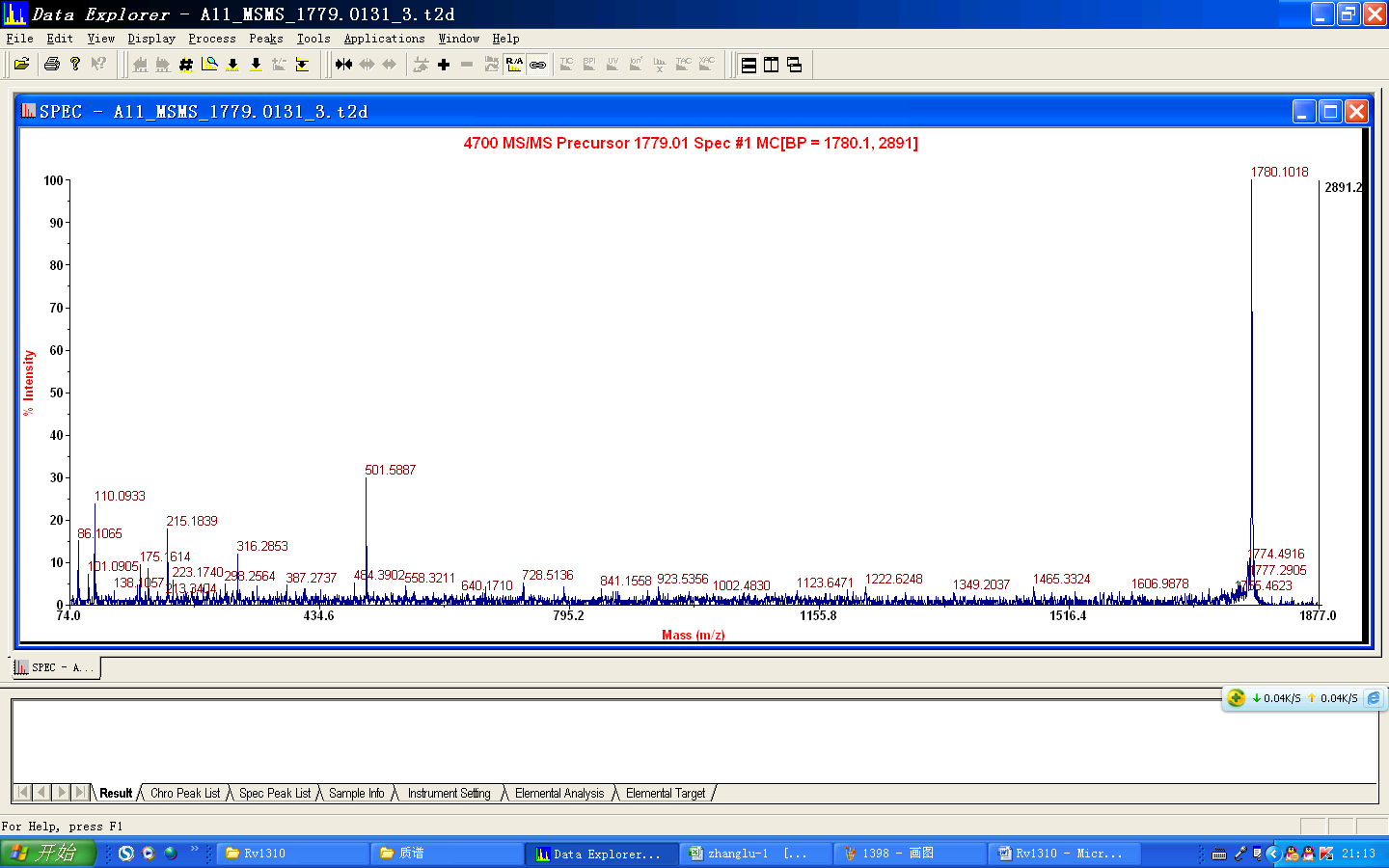


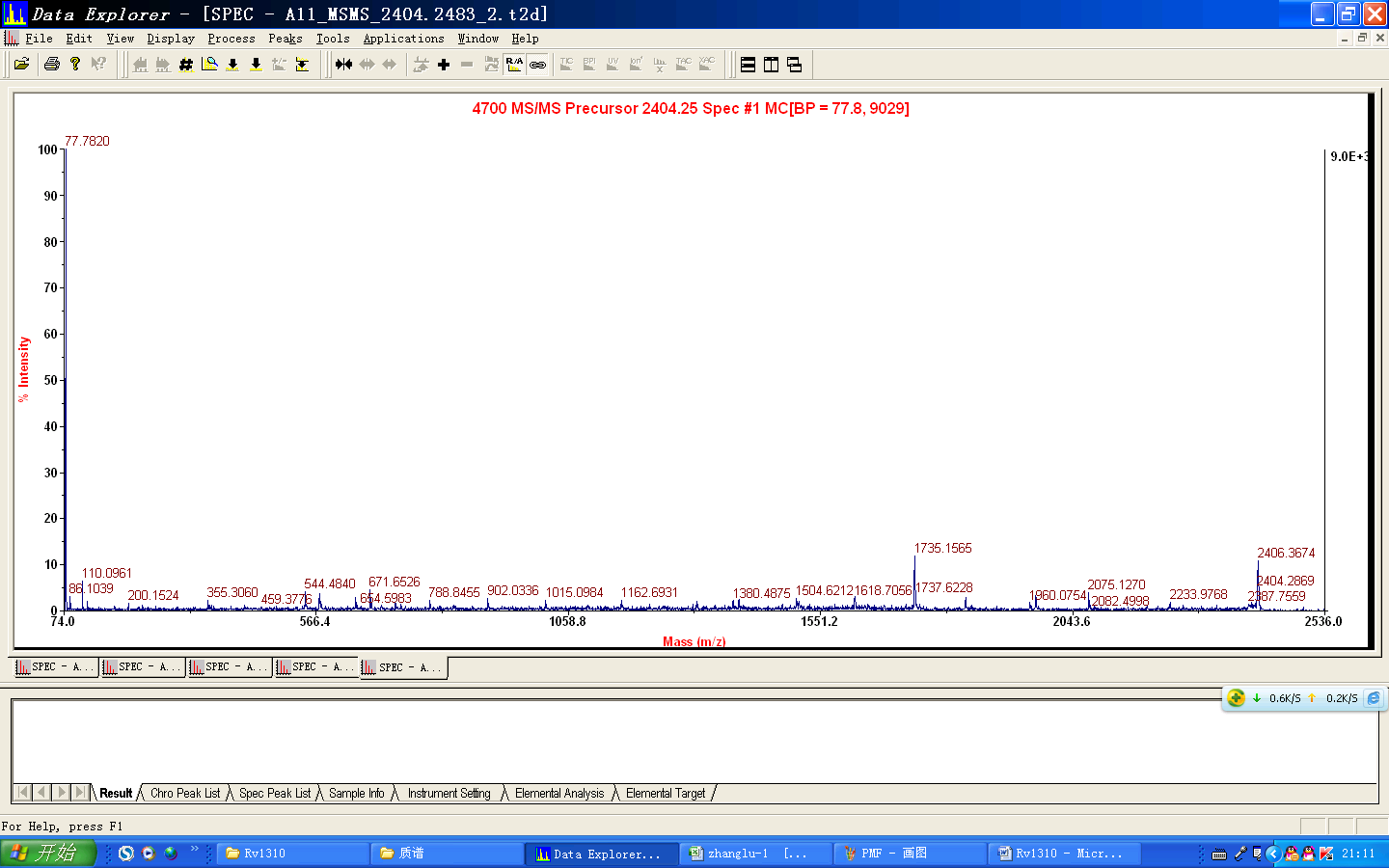

Supplement: Additional file 3 — PMF and MS MS Spectra of Rv1310. [file 1477-5956-10-12-S3.DOC]

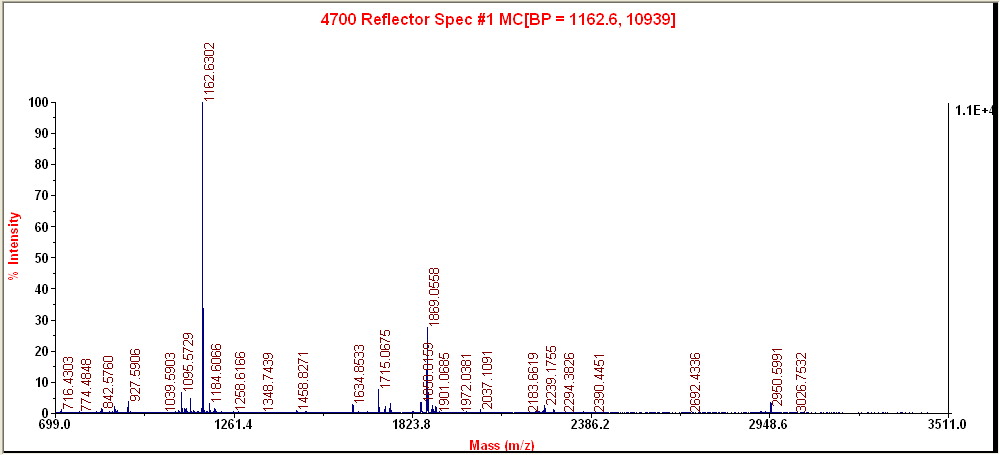


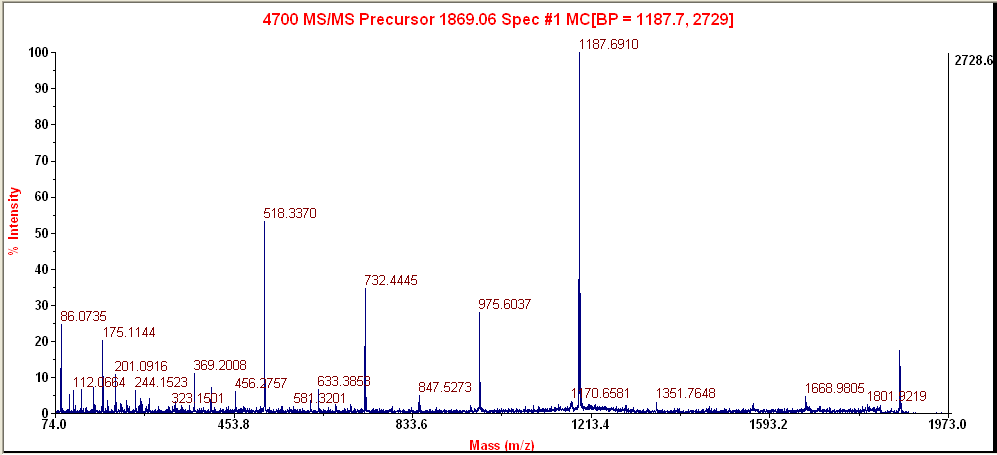


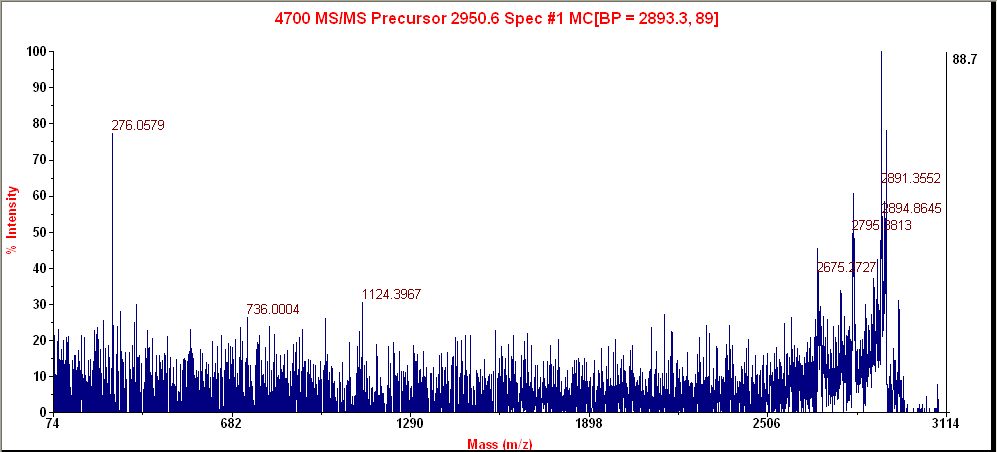


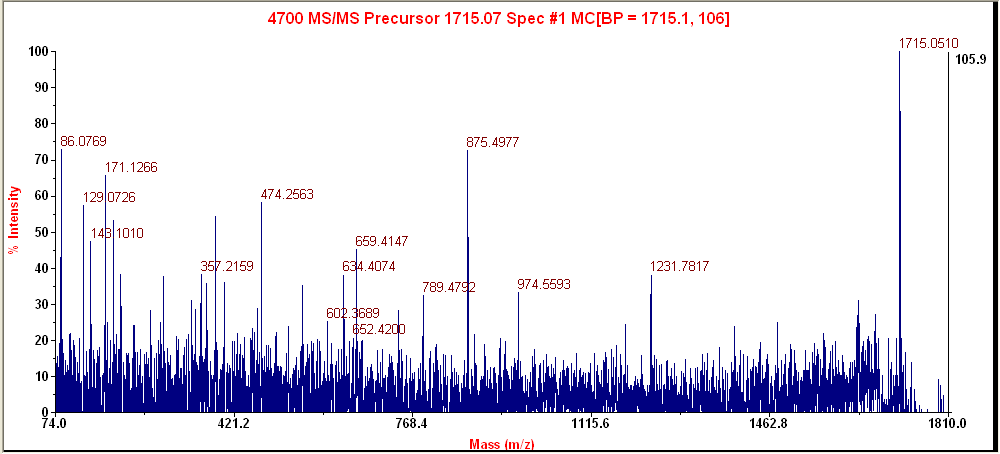


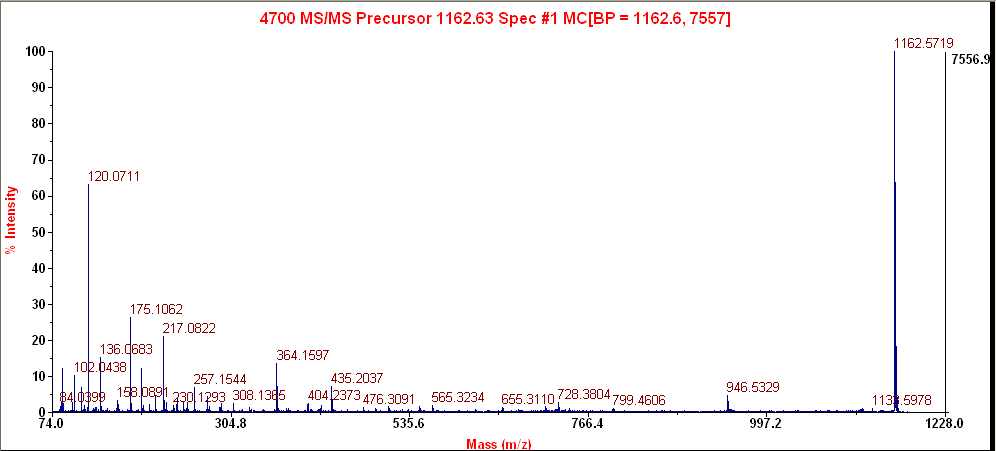

Supplement: Additional file 4 — PMF and MS MS Spectra of Rv2031c. [file 1477-5956-10-12-S4.DOC]

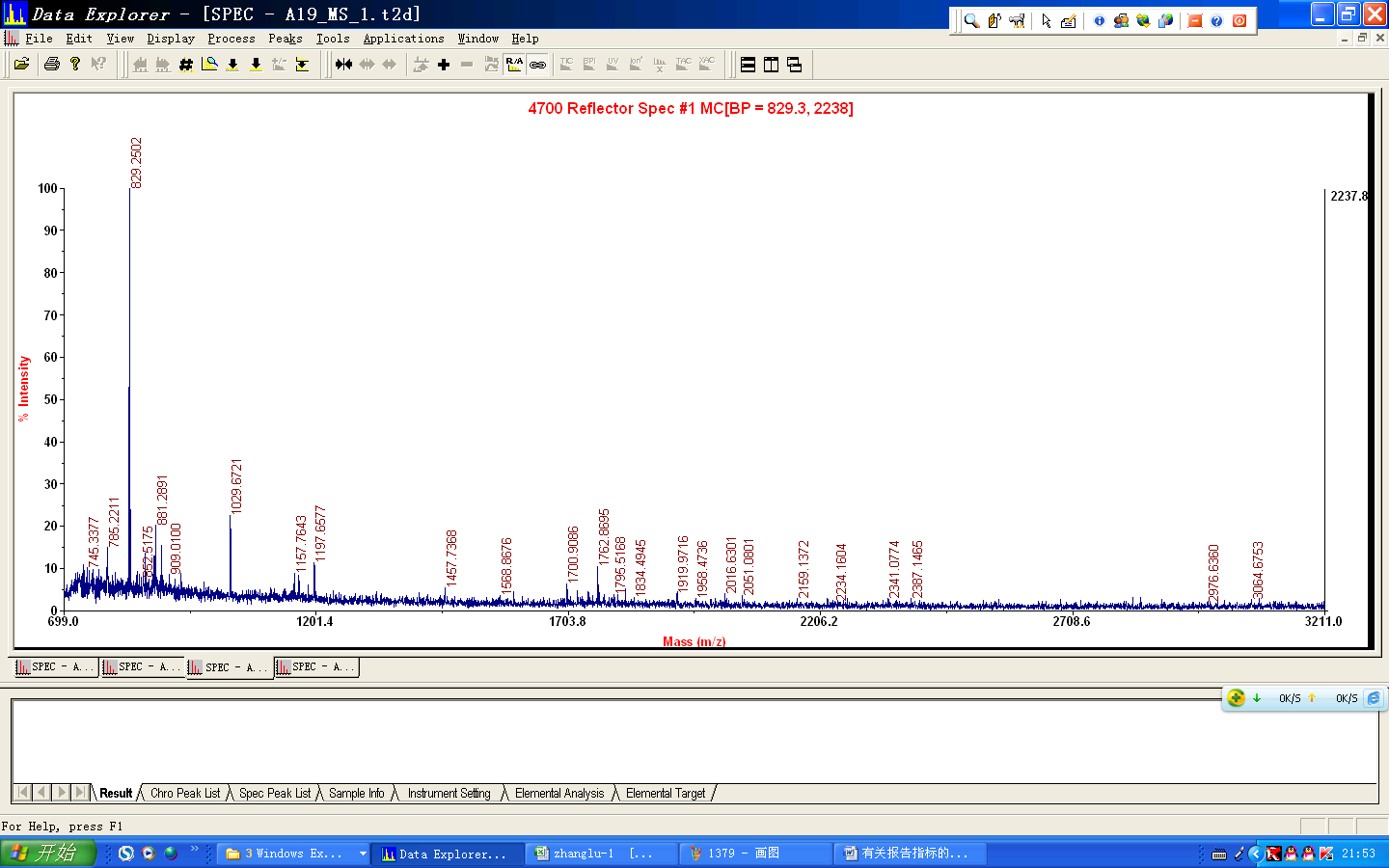


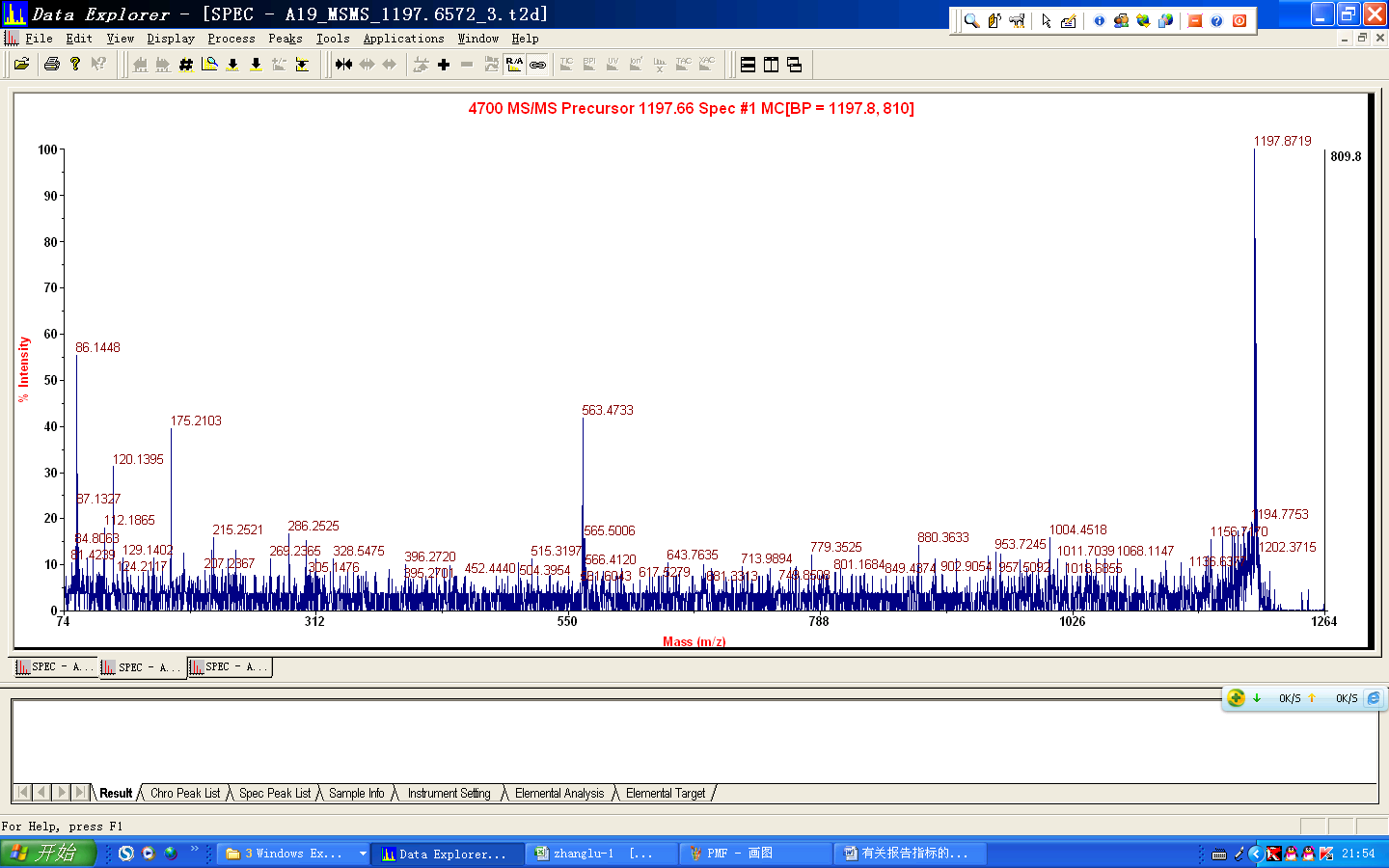


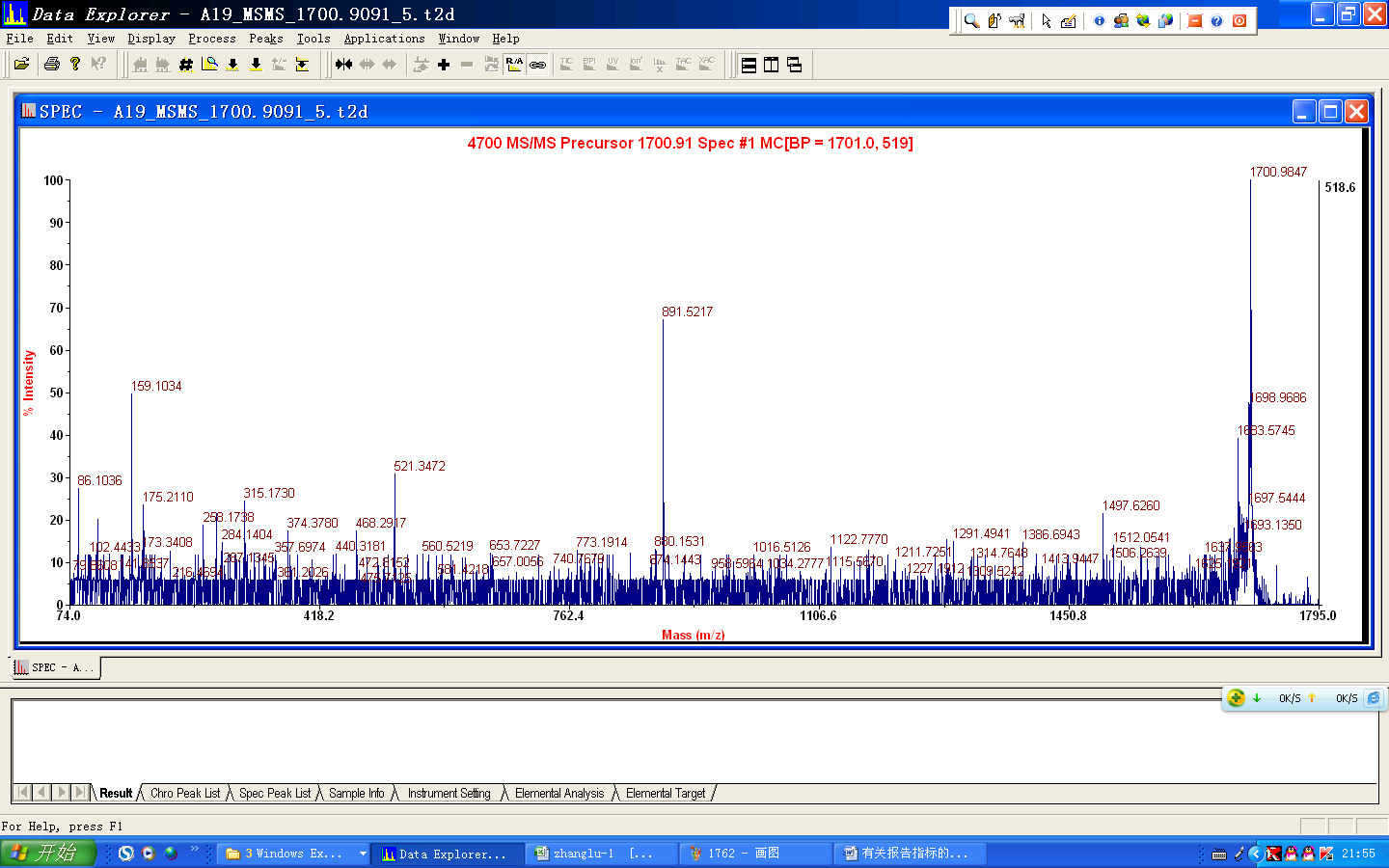


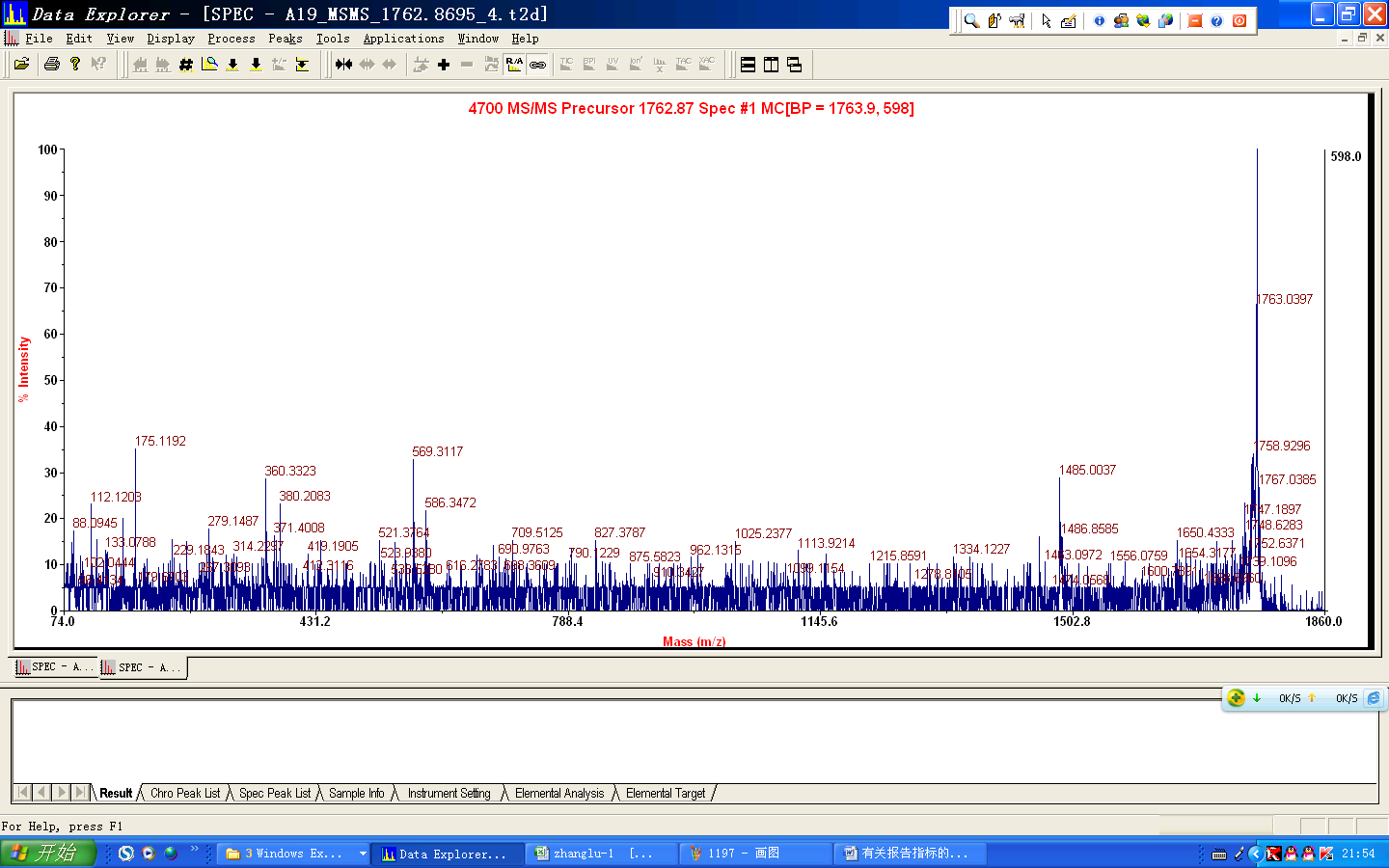

Supplement: Additional file 7 — PMF and MS MS Spectra of Rv3040. [file 1477-5956-10-12-S7.DOC]

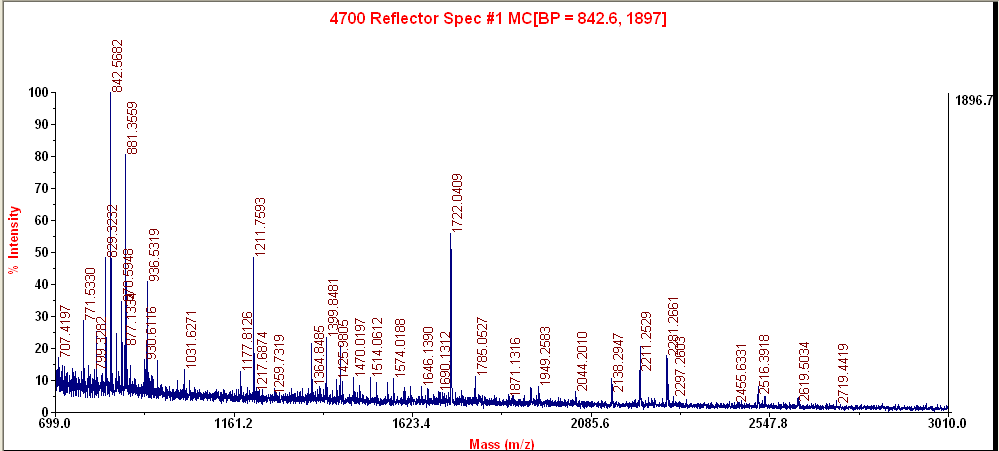


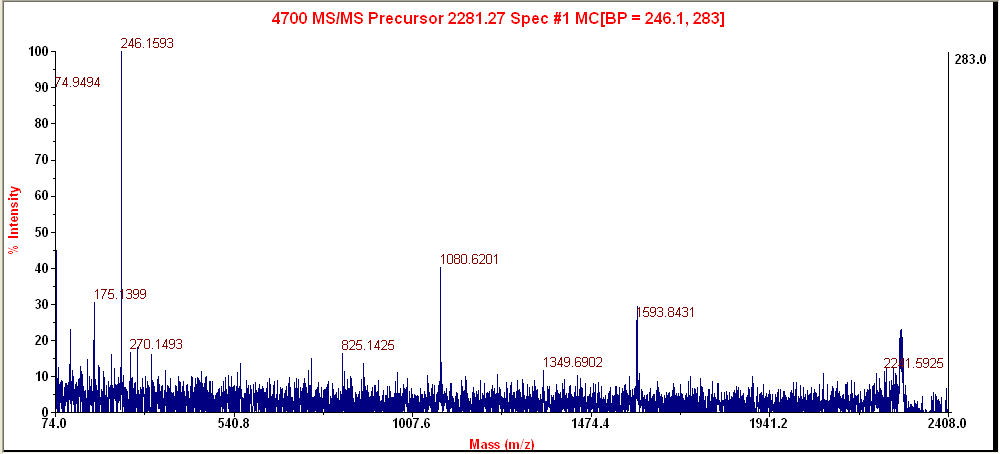


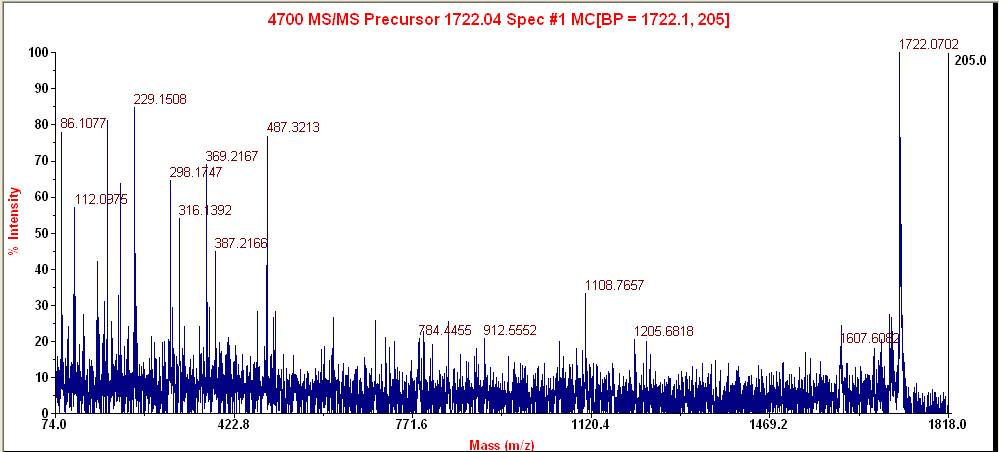


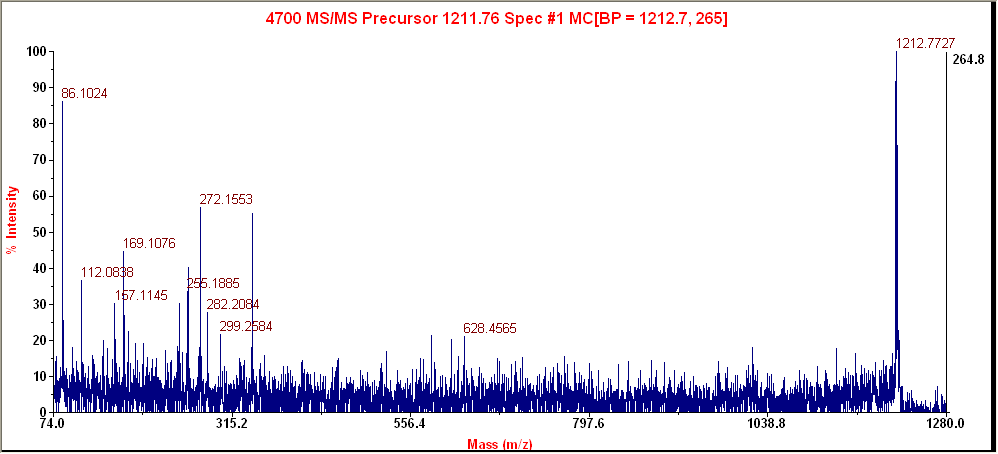


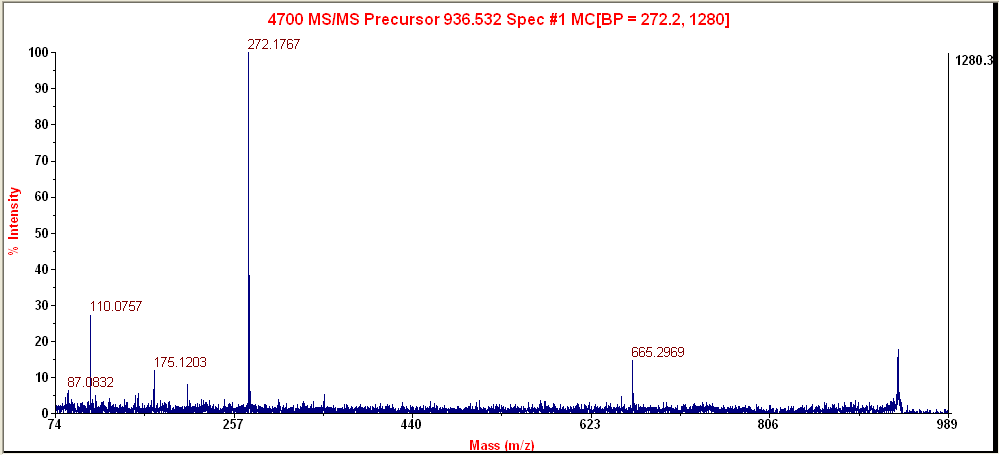

Supplement: Additional file 8 — PMF and MS MS Spectra of Rv3692. [file 1477-5956-10-12-S8.DOC]
