# Supplementary material for: Identification of putative biomarkers for the serodiagnosis of drug-resistant Mycobacterium tuberculosis
Source: Proteome Sci. 2012 Feb 25;10:12. doi: 10.1186/1477-5956-10-12 (PMC3305424; doi:10.1186/1477-5956-10-12)
Supplement: Supplementary file 2 [file 1477-5956-10-12-S1.DOC]

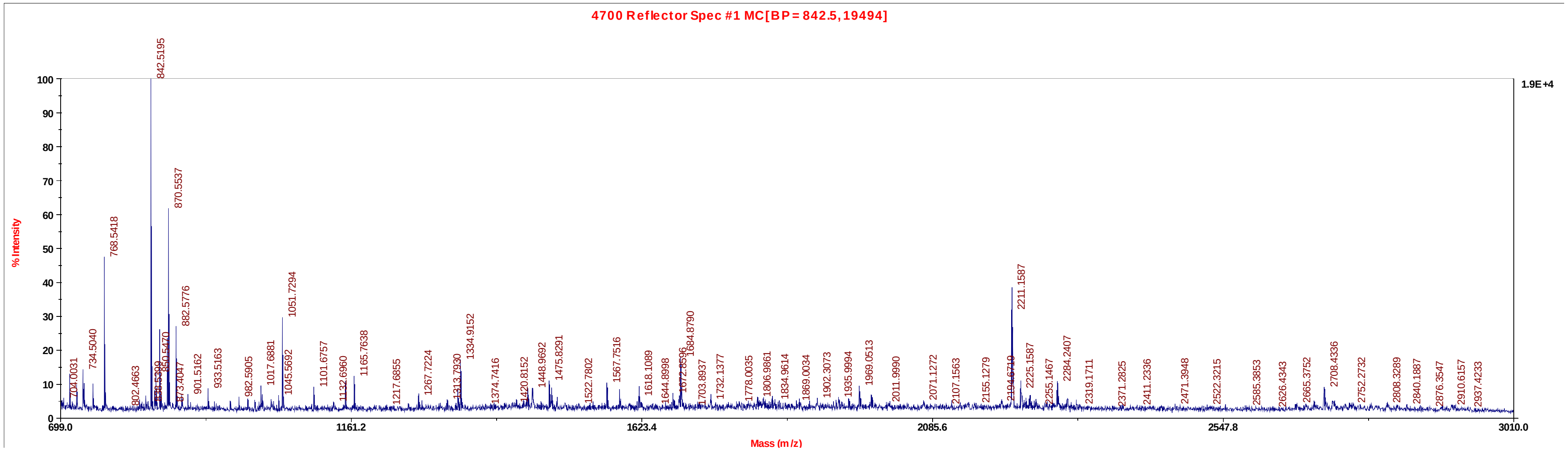
4700 Reflector Spec #1 MC[BP = 842.5, 19494]
% Intensity
Mass (m/z)
1.9E+4
842.5195
870.5537
768.5418
882.5776
734.5040
704.0031
802.4663
901.5162
933.5163
982.5905
1017.6881
1045.5692
1051.7294
1101.6757
1132.6960
1165.7638
1217.6855
1267.7224
1313.7930
1334.9152
1374.7416
1420.8152
1448.9692
1475.8291
1522.7802
1567.7516
1618.1089
1644.8998
1684.8790
1703.8937
1732.1377
1778.0035
1806.9861
1834.9614
1869.0034
1902.3073
1935.9994
1969.0513
2011.9990
2071.1272
2107.1563
2155.1279
2211.1587
2225.1587
2255.1467
2284.2407
2319.1711
2371.2825
2411.2336
2471.3948
2522.3215
2585.3853
2626.4343
2665.3752
2708.4336
2752.2732
2808.3289
2840.1887
2876.3547
2910.6157
2937.4233
699.0
1161.2
1623.4
2085.6
2547.8
3010.0

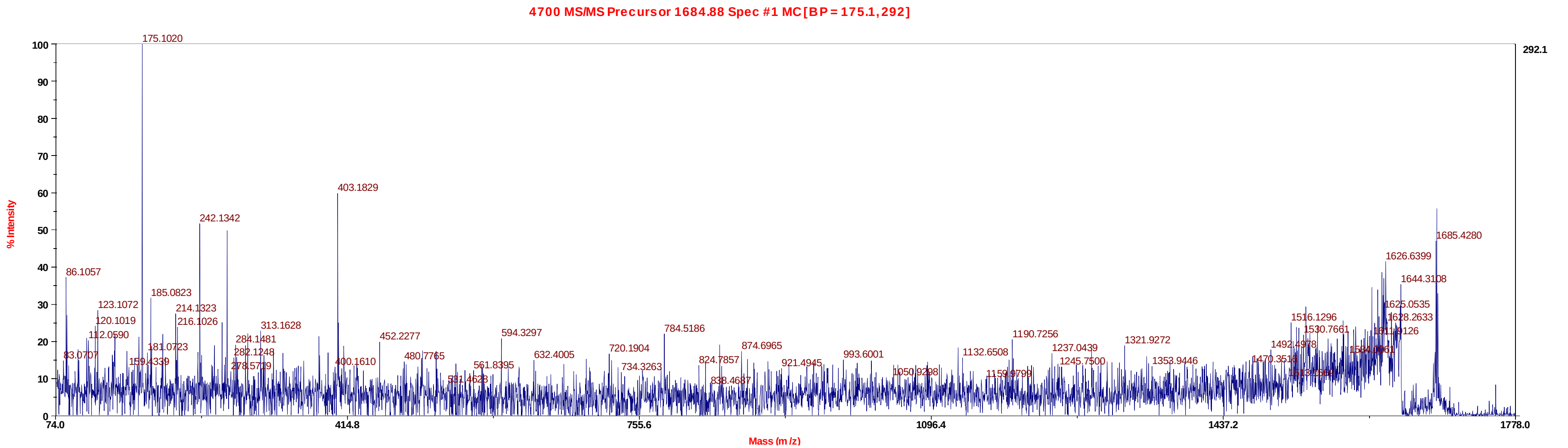
4700 MS/MS Precursor 1684.88 Spec #1 MC[BP = 175.1, 292]
% Intensity
Mass (m/z)
292.1
175.1020
403.1829
242.1342
1685.4280
1626.6399
1644.3108
86.1057
185.0823

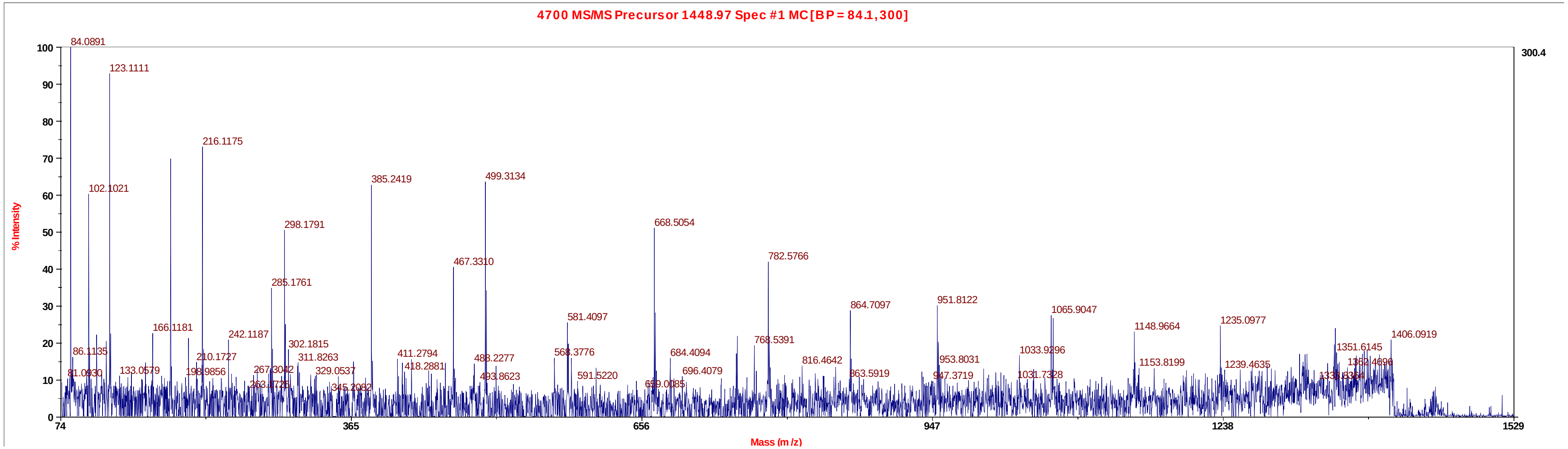
4700 MS/MS Precursor 1448.97 Spec #1 MC[BP = 84.1, 300]
% Intensity
Mass (m/z)
300.4
84.0891
123.1111
216.1175
102.1021
385.2419
499.3134
298.1791
668.5054
467.3310
782.5766
285.1761
581.4097
864.7097
951.8122
1065.9047
1148.9664
1235.0977
166.1181
242.1187
302.1815
768.5391
1406.0919
1351.6145
86.1135
210.1727
311.8263
411.2794
483.2277
568.3776
684.4094
816.4642
953.8031
1033.9296
1153.8199
1239.4635
133.0579
198.9856
267.3042
329.0537
418.2881
696.4079
81.0930
493.8623
591.5220
863.5919
947.3719
1031.7328
263.1726
345.2062
659.0085

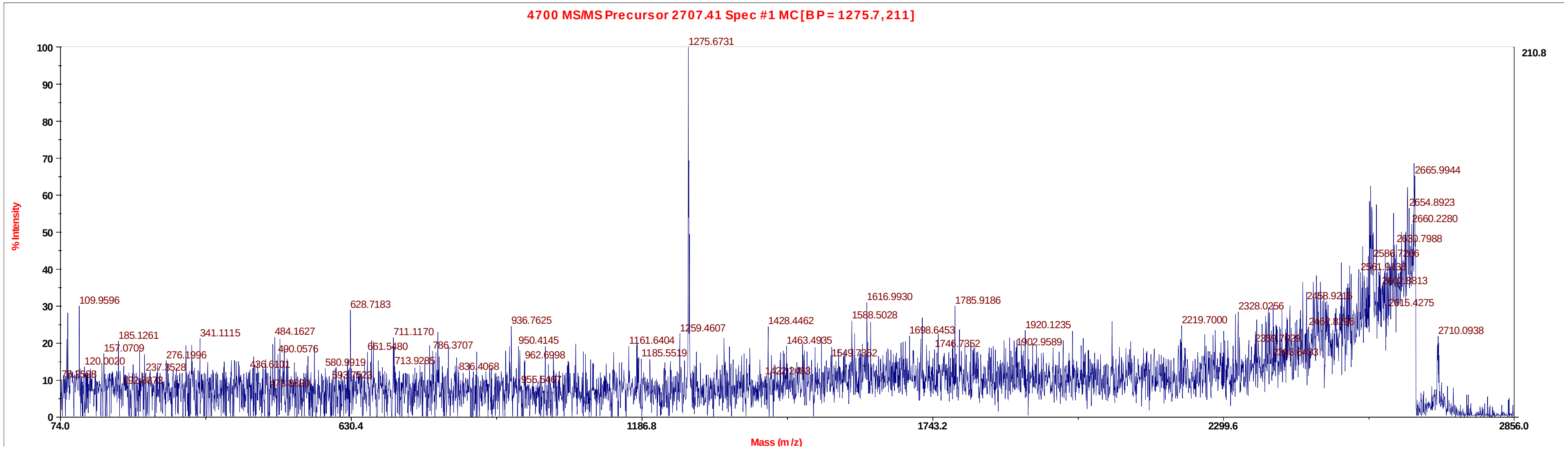
4700 MS/MS Precursor 2707.41 Spec #1 MC[BP = 1275.7, 211]
% Intensity
Mass (m/z)
210.8
1275.6731
2665.9944
2654.8923
2660.2280
2710.0938
109.9596
628.7183
936.7625
1616.9930
1785.9186
1428.4462
1588.5028
2219.7000
2328.0256
1920.1235
1259.4607
341.1115
484.1627
185.1261
711.1170
1161.6404
950.4145
786.3707
661.5480
490.0576
157.0709
962.6998
1185.5519
276.1996
120.0020
713.9285
580.9919
436.6101
836.4068
237.3528
1902.9589
74.0
630.4
1186.8
1743.2
2299.6
2856.0

4700 MS/MS Precursor 1567.75 Spec #1 MC[BP = 120.1, 205]

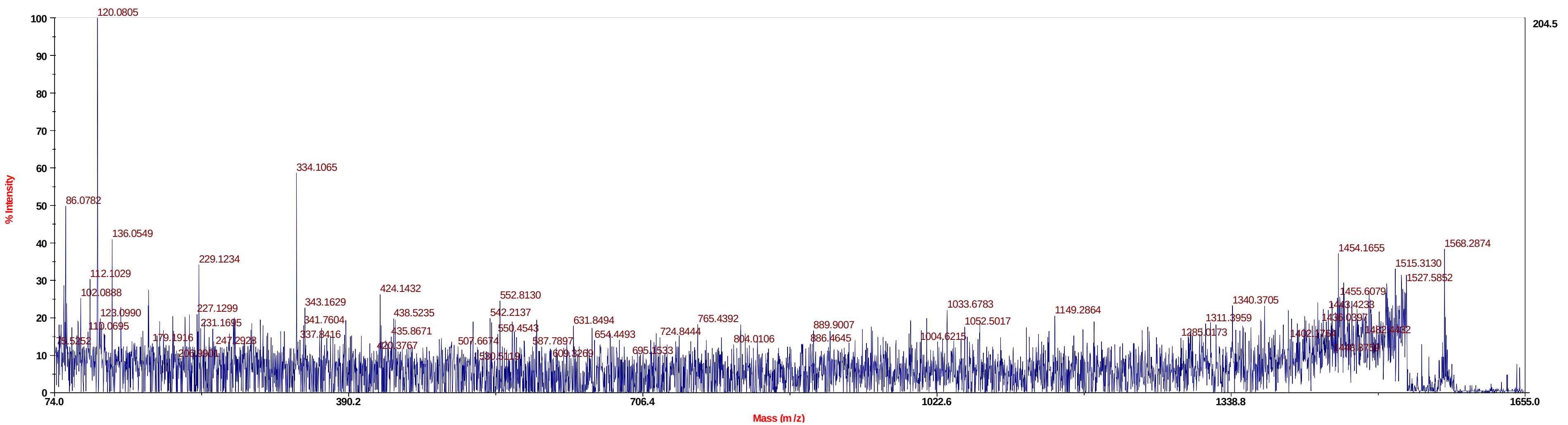

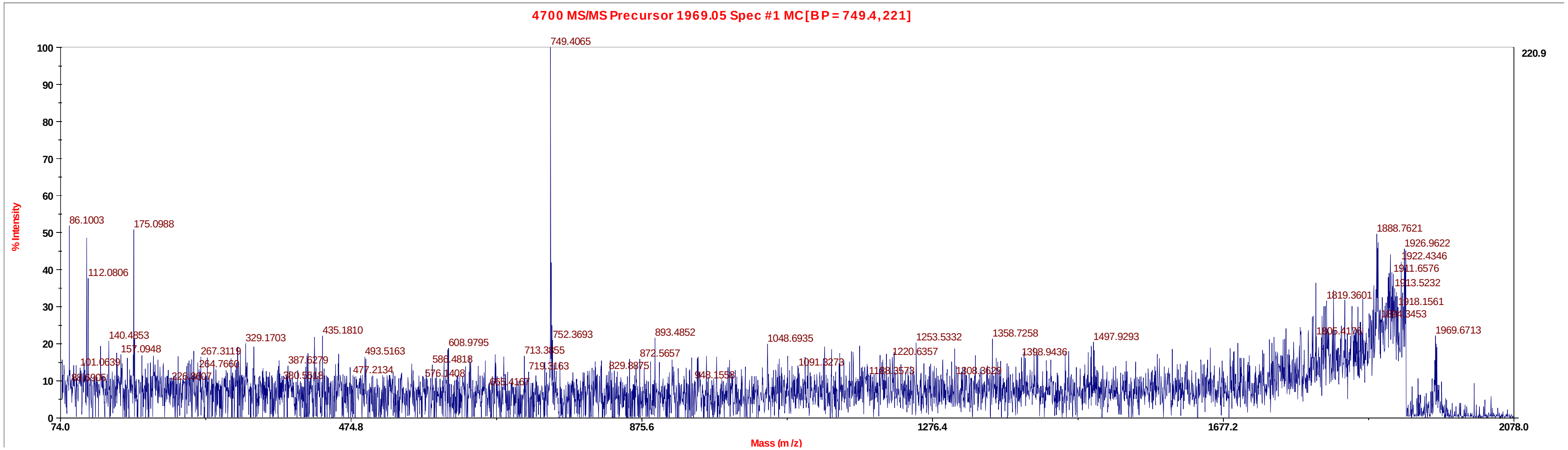
4700 MS/MS Precursor 1969.05 Spec #1 MC[BP = 749.4, 221]
% Intensity
Mass (m/z)
220.9
749.4065
86.1003
175.0988
112.0806
140.4853
157.0948
101.0639
329.1703
435.1810
493.5163
608.9795
752.3693
893.4852
1048.6935
1253.5332
1358.7258
1497.9293
1888.7621
1926.9622
1922.4346
1911.6576
1819.3601
1918.1561
1969.6713
74.0
474.8
875.6
1276.4
1677.2
2078.0
