# Supplementary material for: Identification of putative biomarkers for the serodiagnosis of drug-resistant Mycobacterium tuberculosis
Source: Proteome Sci. 2012 Feb 25;10:12. doi: 10.1186/1477-5956-10-12 (PMC3305424; doi:10.1186/1477-5956-10-12)
Supplement: Supplementary file 3 [file 1477-5956-10-12-S5.DOC]

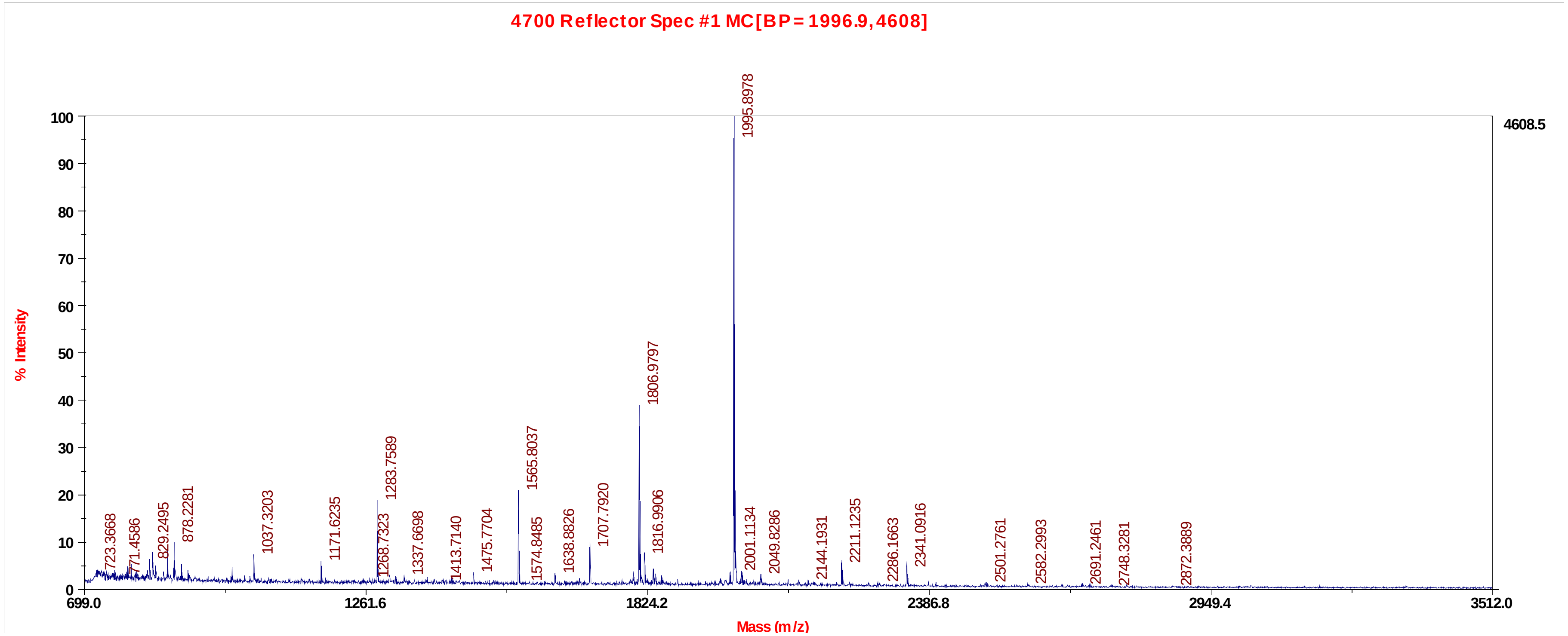

4700 Reflector Spec #1 MC[BP = 1996.9, 4608]
% Intensity
Mass (m/z)
4608.5
0
10
20
30
40
50
60
70
80
90
100
699.0
1261.6
1824.2
2386.8
2949.4
3512.0
1995.8978
1806.9797
1565.8037
1283.7589
723.3668
771.4586
829.2495
878.2281
1037.3203
1171.6235
1268.7323
1337.6698
1413.7140
1475.7704
1574.8485
1638.8826
1707.7920
1816.9906
2001.1134
2049.8286
2144.1931
2211.1235
2286.1663
2341.0916
2501.2761
2582.2993
2691.2461
2748.3281
2872.3889

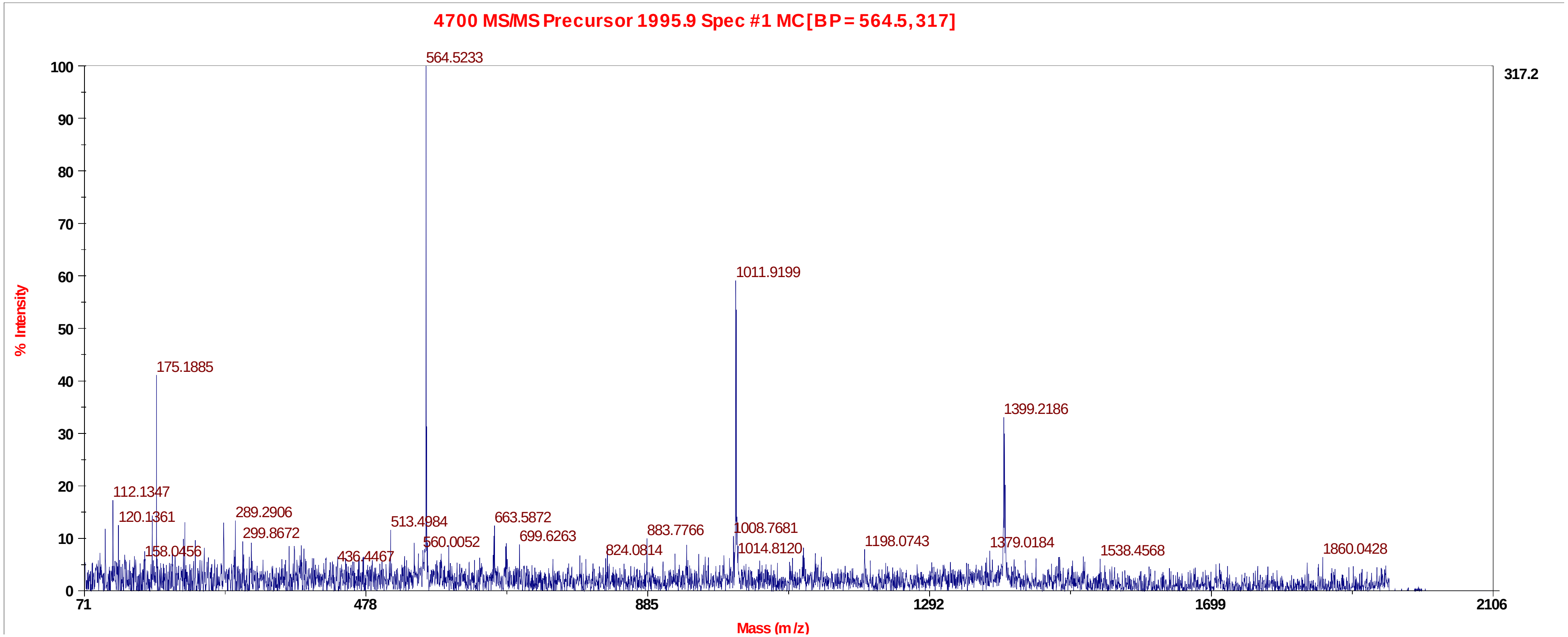
4700 MS/MS Precursor 1995.9 Spec #1 MC[BP = 564.5, 317]
% Intensity
Mass (m/z)
317.2
564.5233
1011.9199
175.1885
1399.2186
112.1347
120.1361
158.0456
289.2906
299.8672
436.4467
513.4984
560.0052
663.5872
699.6263
824.0814
883.7766
1008.7681
1014.8120
1198.0743
1379.0184
1538.4568
1860.0428
71
478
885
1292
1699
2106
0
10
20
30
40
50
60
70
80
90
100
