# Supplementary material for: Identification of putative biomarkers for the serodiagnosis of drug-resistant Mycobacterium tuberculosis
Source: Proteome Sci. 2012 Feb 25;10:12. doi: 10.1186/1477-5956-10-12 (PMC3305424; doi:10.1186/1477-5956-10-12)
Supplement: Supplementary file 4 [file 1477-5956-10-12-S6.DOC]

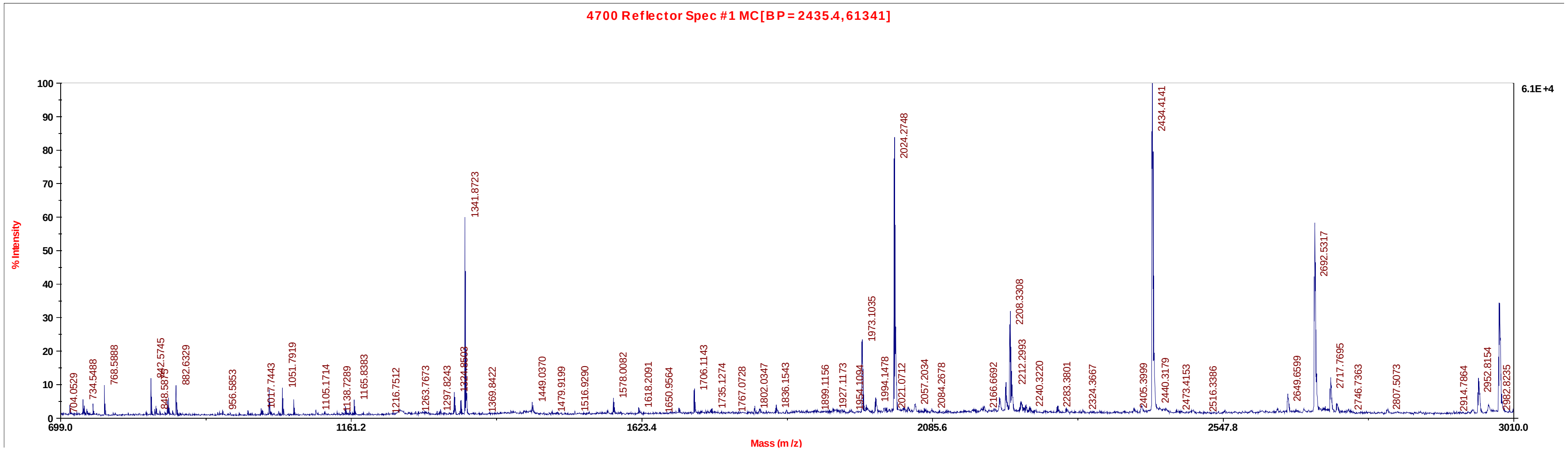
4700 Reflector Spec #1 MC[BP = 2435.4, 61341]
% Intensity
Mass (m/z)
6.1E+4
1341.8723
2024.2748
2434.4141
2692.5317
2208.3308
1973.1035

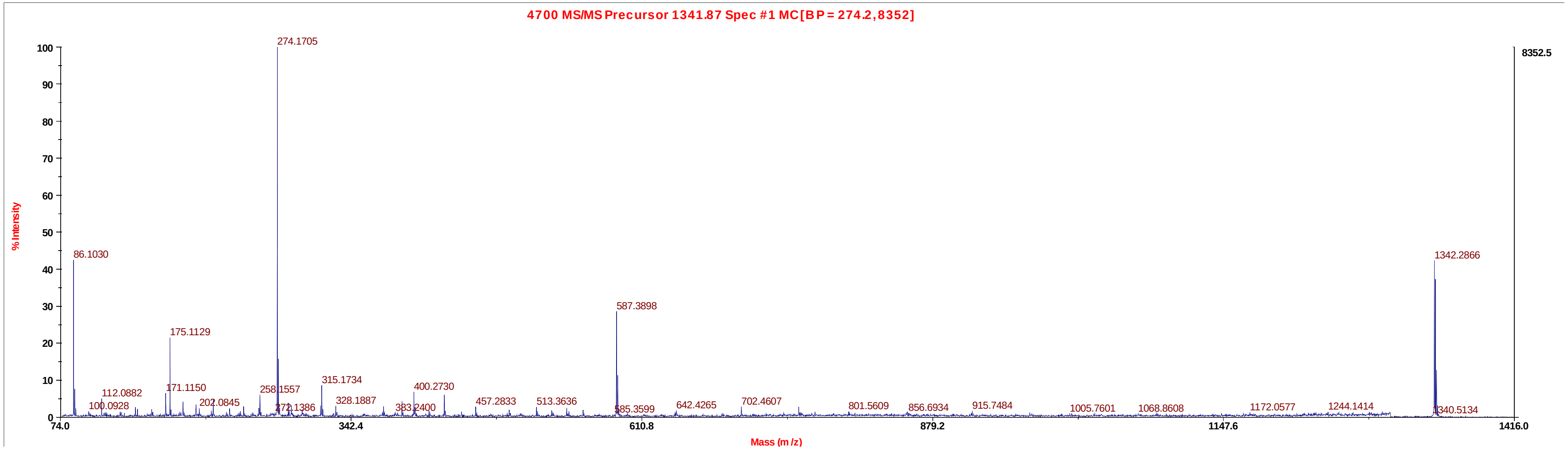

4700 MS/MS Precursor 1341.87 Spec #1 MC[BP = 274.2, 8352]
% Intensity
Mass (m/z)
8352.5
274.1705
86.1030
1342.2866
587.3898
175.1129
112.0882
171.1150
258.1557
315.1734
400.2730
100.0928
202.0845
272.1386
328.1887
383.2400
457.2833
513.3636
585.3599
642.4265
702.4607
801.5609
856.6934
915.7484
1005.7601
1068.8608
1172.0577
1244.1414
1340.5134
74.0
342.4
610.8
879.2
1147.6
1416.0

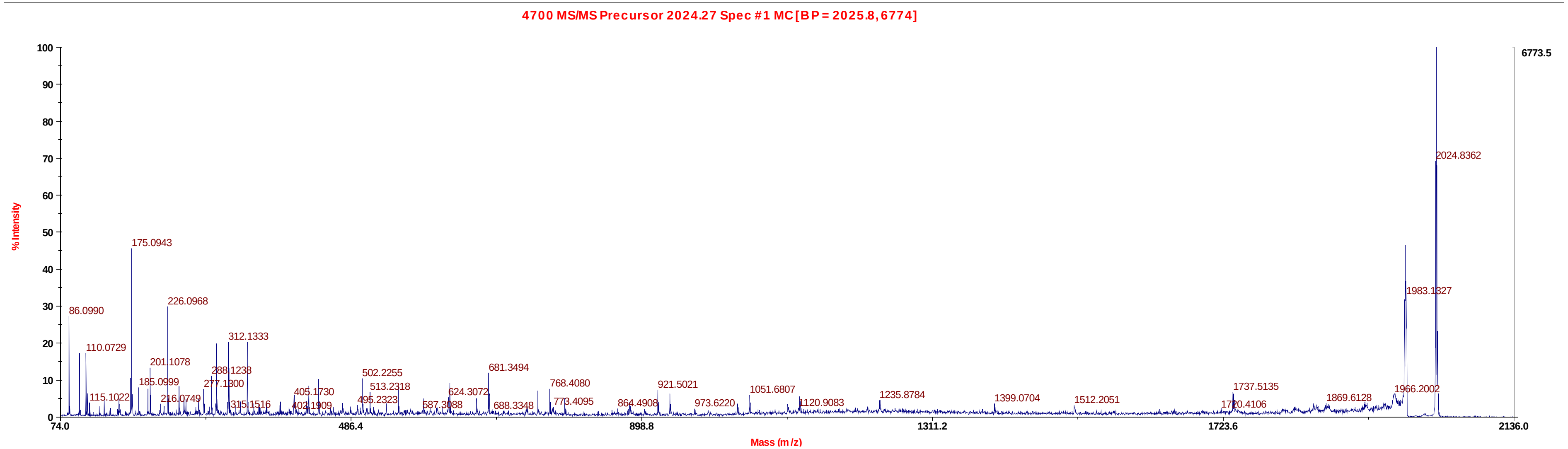
4700 MS/MS Precursor 2024.27 Spec #1 MC[BP = 2025.8, 6774]
% Intensity
Mass (m/z)
6773.5
2024.8362
1983.1327
175.0943
226.0968
86.0990
110.0729
312.1333
201.1078
288.1238
185.0999
277.1300
115.1022
216.0749
315.1516
405.1730
402.1909
502.2255
513.2318
495.2323
587.3088
624.3072
681.3494
688.3348
768.4080
773.4095
864.4908
921.5021
973.6220
1051.6807
1120.9083
1235.8784
1399.0704
1512.2051
1737.5135
1720.4106
1869.6128
1966.2002

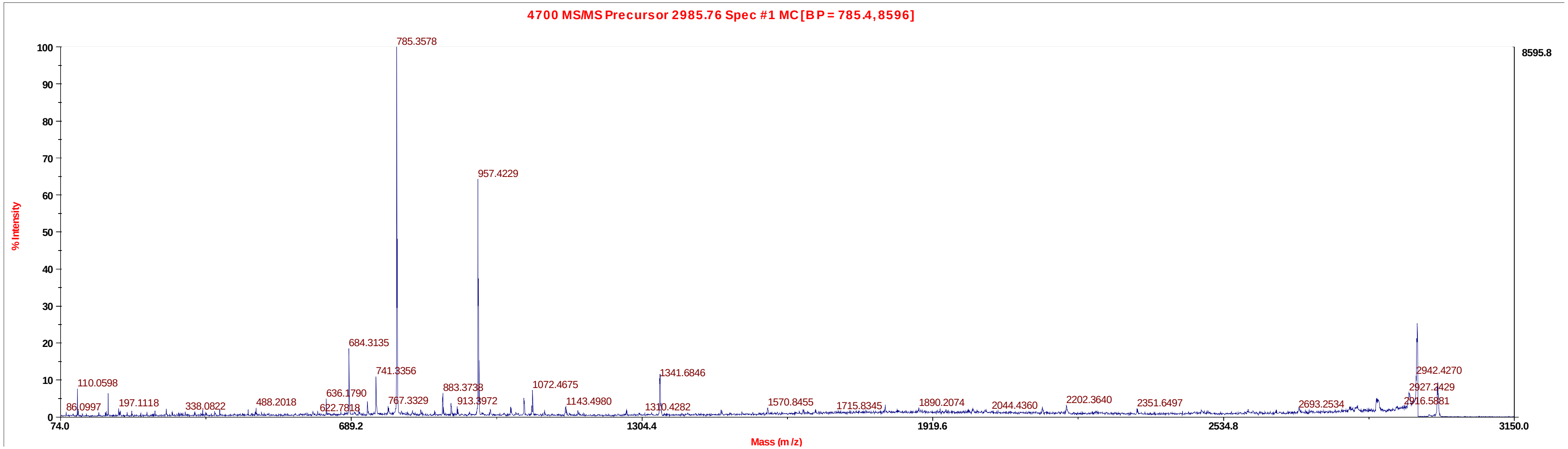
4700 MS/MS Precursor 2985.76 Spec #1 MC[BP = 785.4, 8596]
% Intensity
Mass (m/z)
8595.8
785.3578
957.4229
684.3135
741.3356
110.0598
1341.6846
2942.4270
2927.2429
2916.5881
1072.4675
883.3738
636.1790
767.3329
913.3972
1143.4980
1310.4282
1570.8455
1715.8345
1890.2074
2044.4360
2202.3640
2351.6497
2693.2534
86.0997
197.1118
338.0822
488.2018
622.7818
74.0
689.2
1304.4
1919.6
2534.8
3150.0

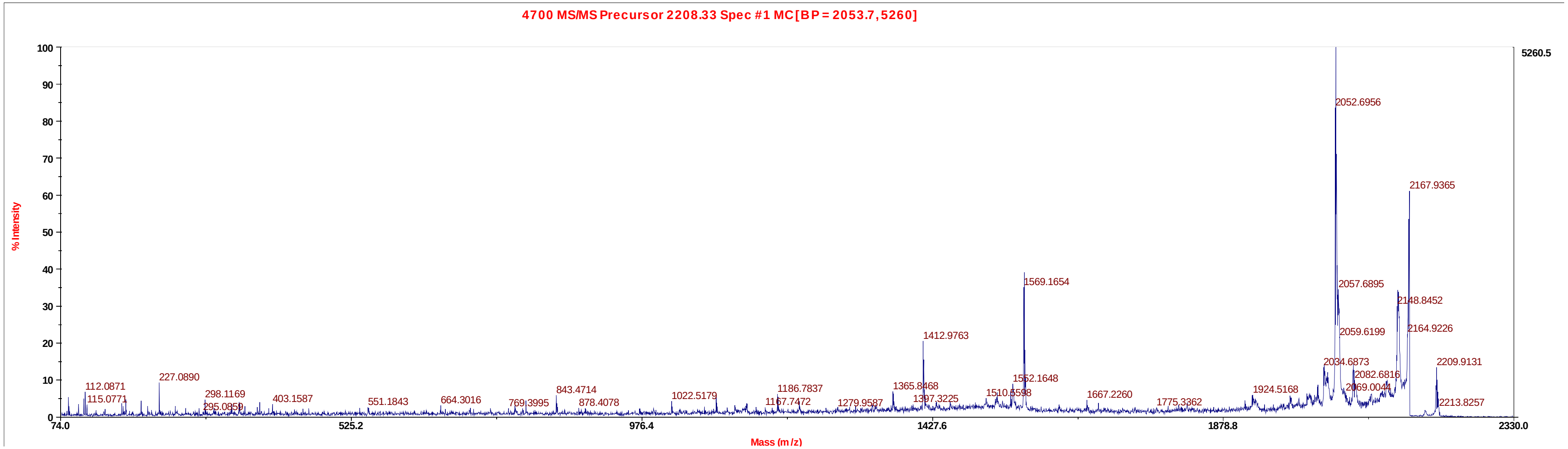
4700 MS/MS Precursor 2208.33 Spec #1 MC[BP = 2053.7, 5260]
% Intensity
Mass (m/z)
5260.5
100
90
80
70
60
50
40
30
20
10
0
74.0
525.2
976.4
1427.6
1878.8
2330.0
2052.6956
2167.9365
1569.1654
2057.6895
2148.8452
1412.9763
2059.6199
2164.9226
2034.6873
2209.9131
227.0890
112.0871
1186.7837
1365.8468
1552.1648
2082.6816
843.4714
298.1169
1022.5179
1924.5168
1510.5598
2069.0044
115.0771
403.1587
1667.2260
551.1843
664.3016
1397.3225
1167.7472
295.0859
769.3995
878.4078
1279.9587
1775.3362
2213.8257

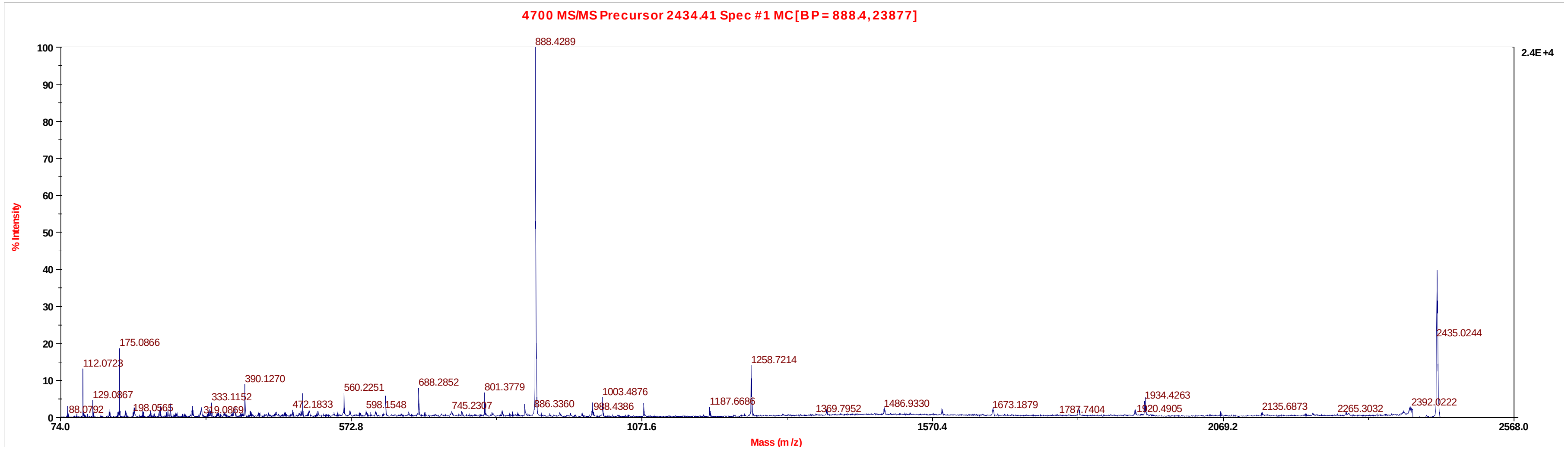
4700 MS/MS Precursor 2434.41 Spec #1 MC[BP = 888.4, 23877]
% Intensity
Mass (m/z)
2.4E+4
888.4289
2435.0244
1258.7214
175.0866
112.0723
390.1270
688.2852
560.2251
801.3779
1003.4876
1934.4263
129.0867
333.1152
198.0565
88.0792
319.0869
472.1833
598.1548
745.2307
886.3360
988.4386
1187.6686
1369.7952
1486.9330
1673.1879
1787.7404
1920.4905
2135.6873
2265.3032
2392.0222
74.0
572.8
1071.6
1570.4
2069.2
2568.0

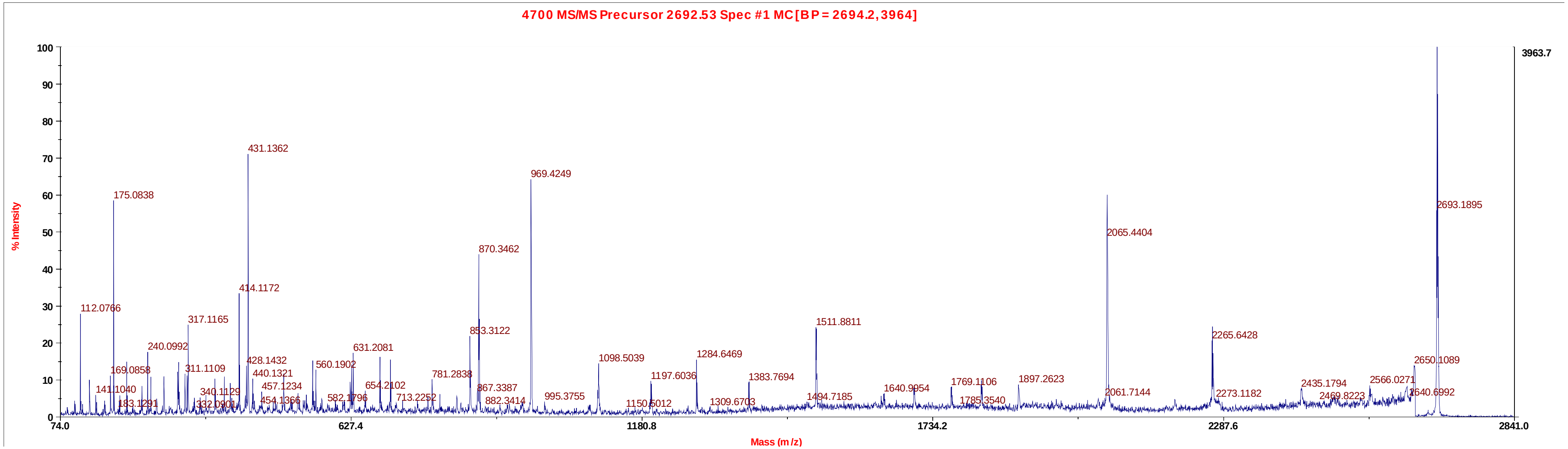
4700 MS/MS Precursor 2692.53 Spec #1 MC[BP = 2694.2, 3964]
% Intensity
Mass (m/z)
3963.7
175.0838
431.1362
969.4249
2065.4404
2693.1895
870.3462
414.1172
112.0766
317.1165
853.3122
1511.8811
2265.6428
240.0992
631.2081
1098.5039
1284.6469
2650.1089
169.0858
311.1109
428.1432
560.1902
440.1321
781.2838
1197.6036
1383.7694
1769.1106
1897.2623
2435.1794
2566.0271
141.1040
340.1129
457.1234
654.2102
867.3387
1640.9954
2061.7144
2273.1182
2640.6992
2469.8223
995.3755
1494.7185
713.2252
582.1796
454.1366
882.3414
1785.3540
183.1291
332.0901
1150.6012
1309.6703
